# Selective decoupling of IgG1 binding to viral Fc receptors restores antibody-mediated NK cell activation against HCMV

**DOI:** 10.1016/j.celrep.2025.116593

**Published:** 2025-12-03

**Authors:** Ahlam N. Qerqez, Katja Hoffmann, Alison G. Lee, Sumit Pareek, Kelli Hager, Akaash K. Mishra, George Delidakis, Kirsten Bentley, Lauren Kerr-Jones, Mica Cabrera, Truong Nguyen, Rebecca L. Göttler, Amjad Chowdhury, Philipp L. Kolb, Hartmut Hengel, George Georgiou, Jason S. McLellan, Richard J. Stanton, Annalee W. Nguyen, Jennifer A. Maynard

**Affiliations:** 1 Department of Chemical Engineering, University of Texas at Austin, Austin, TX 78712, USA; 2 Department of Molecular Biosciences, University of Texas at Austin, Austin, TX 78712, USA; 3 Division of Infection and Immunology, School of Medicine, Cardiff University, Cardiff CF14 4XN, UK; 4 Institute of Virology, University Medical Center, and Faculty of Medicine, University of Freiburg, 79104 Freiburg, Germany; 5 LaMontaigne Center for Infectious Diseases, University of Texas at Austin, Austin, TX 78712, USA; 6 These authors contributed equally; 7 Lead contact

## Abstract

Antibodies binding cell-surface antigens to activate cellular immunity are an important mechanism of anti-viral protection, yet antibodies targeting cells infected by human cytomegalovirus (HCMV) exhibit limited efficacy. This is due to HCMV immune evasion mechanisms, including viral receptors (vFcγRs) that bind human immunoglobulin G Fc domains to inhibit host Fcγ receptor activation and impair Fc-mediated immune functions. Here, we biochemically characterize two conserved vFcγRs, gp34 and gp68, and map their Fc binding sites. We then engineer Fc variants to retain binding to host Fc receptors CD16A and FcRn but exhibit markedly reduced gp34/gp68 interactions. Antibodies targeting the gB fusogen with engineered Fc domains are not internalized by infected cells, mediate enhanced immune cell activation, and limit viral spread in HCMV-infected fibroblasts more effectively than antibodies with wild-type Fc. This work demonstrates a strategy to enhance therapeutic antibody control of HCMV infections and other herpesvirus infections with similar immune-evasion mechanisms.

## In brief

Antibodies targeting human cytomegalovirus (HCMV) are undermined by viral immune evasion strategies, including capture of the Fc. Qerqez et al. engineer human Fc variants with markedly reduced binding to two viral Fc receptors. Engineered antibodies mediate enhanced CD16A-activation against HCMV-infected fibroblasts and limit viral spread more effectively than non-engineered antibodies.

## Graphical abstract

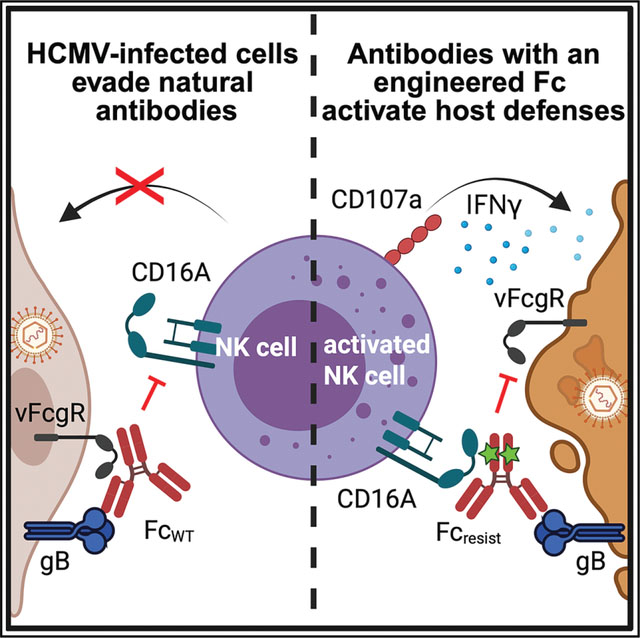

## INTRODUCTION

Human cytomegalovirus (HCMV) infection is widespread and increases with age,^1,2^ with up to ~60% of the US population exhibiting seropositivity by the age of 50.^3^ HCMV is usually asymptomatic in immunocompetent individuals, but can present as significant disease in those with compromised immune systems, including bone marrow and organ-transplant patients and neonates. HCMV is also the leading infectious cause of congenital birth defects,^4^ with transmission occurring in 0.5%–2% of pregnancies.^4–6^ The challenges of using toxic anti-viral therapeutics in patients with significant comorbidities^7^ have spurred interest in antibody-based treatments. However, administration of intravenous immunoglobulin containing high-titer HCMV neutralizing antibodies to pregnant women does not protect the fetus,^8^ nor is it the standard treatment for transplant patients.^9^ Similarly, four neutralizing antibodies targeting the fusogenic glycoprotein gB or entry glycoprotein gH (MSL109 and its high-affinity variants RG7667, CSJ148, and NPC-21) were not effective in phase II trials.^10^ These efforts focused on neutralizing antibodies, suggesting this activity may be insufficient to control HCMV *in vivo*.

Since HCMV disseminates as a highly cell-associated virus,^11^ therapeutics that also target infected cells may provide greater efficacy. Antibodies binding viral antigens on the infected cell surface can suppress disease by recruiting immune cells to eliminate infected cells. Accumulating evidence indicates that these Fc effector functions are a crucial component of protection from HCMV. Studies using sera from gB vaccinees and seropositive women revealed strong correlations between protection and Fc-mediated antibody-dependent cellular cytotoxicity (ADCC) and phagocytosis (ADCP).^12–14^ However, these antibody activities are undermined by multiple HCMV viral proteins that inhibit natural killer (NK) cell activities,^15,16^ including viral Fcγ receptors (vFcγRs). These viral proteins bind antibody Fc domains to antagonize the activation of host Fcγ receptors, including CD16A, which mediates ADCC by NK cells.

HCMV expresses four putative vFcγRs on the surface of infected cells and in the virion envelope.^17–22^ Their presence is conserved across HCMV clinical isolates, with genes *RL11* and *UL118-UL119* (encoding gp34 and gp68, respectively) showing little sequence variation, whereas *RL12* and *RL13* are highly divergent.^18^ The two conserved vFcγRs, gp34 and gp68, bind distinct Fc epitopes and cooperate to internalize and clear the surface of infected cells of antibody/viral glycoprotein complexes, thereby limiting antibody-mediated effector functions.^23^ Gp68 appears to have compromised a phase II clinical trial for antibody MSL-109, which was internalized by vFcγRs and incorporated into new virions.^24,25^ Conversely, removal of the three validated vFcγRs from a rhesus CMV (RhCMV) strain resulted in stronger host Fc receptor functions *in vitro* and a shorter duration of HCMV DNAemia *in vivo*.^26^ Viral control coincided with an increase in anti-HCMV antibody titer, suggesting that antibody Fc mechanisms played a larger role in the absence of vFcγRs. These results highlight the importance of Fc-mediated effector functions in viral control and the potential for vFcγRs to dampen these responses.

The conservation and complementary functions of gp34 and gp68 led us to speculate that disrupting Fc capture by these vFcγRs would restore CD16A activation by anti-HCMV antibodies against viral targets such as gB. Accordingly, we defined the interactions between these two vFcγRs and human immunoglobulin G1 (IgG1) and used this information to guide the engineering of Fc variants that resist vFcγR capture while retaining host-FcγR interactions. Fifty years after antibody capture by HCMV-infected cells was first described,^27^ development of engineered Fcs with reduced vFcγR affinities provides insight into this highly evolved immune-evasion mechanism and suggests strategies to design potent anti-HCMV antibody therapeutics.

## RESULTS

### HCMV vFcγRs antagonize CD16A activation by internalizing human anti-gB antibodies

We first aimed to define the impact of the vFcγRs on CD16A activation and antibody internalization using human MRC-5 fibroblasts infected with the common HCMV strain AD169 or an isogenic variant lacking expression of the three functional genes encoding vFcγRs (*RL11*, *RL12*, and *UL119–118)* called Δ3. *RL13* was not considered because its expression is rapidly lost during *in vitro* culture. HMCV gB and the vFcγRs have similar expression kinetics with cell surface levels peaking 72–96 h post-infection (hpi), indicating that gB-binding antibodies may be maximally impacted by vFcγRs at this time.^15,20,28,29^ We selected the gB-specific antibody SM5–1,^30^ which binds pre- and post-fusion gB conformations on infected cells and virions but poorly activates NK cells against HCMV-infected cells.^12^ The SM5–1 Fab with human C_H_1 and kappa constant domains was expressed with a wild-type human IgG1 Fc or a control mouse IgG2a Fc (SM5–1-mFc), which does not bind vFcγRs.^31^ Additionally, we used Cytotect, a commercial high-titer HCMV polyclonal human immunoglobulin known to mediate ADCC as a positive control.^17,23,32^

**Figure 1.**
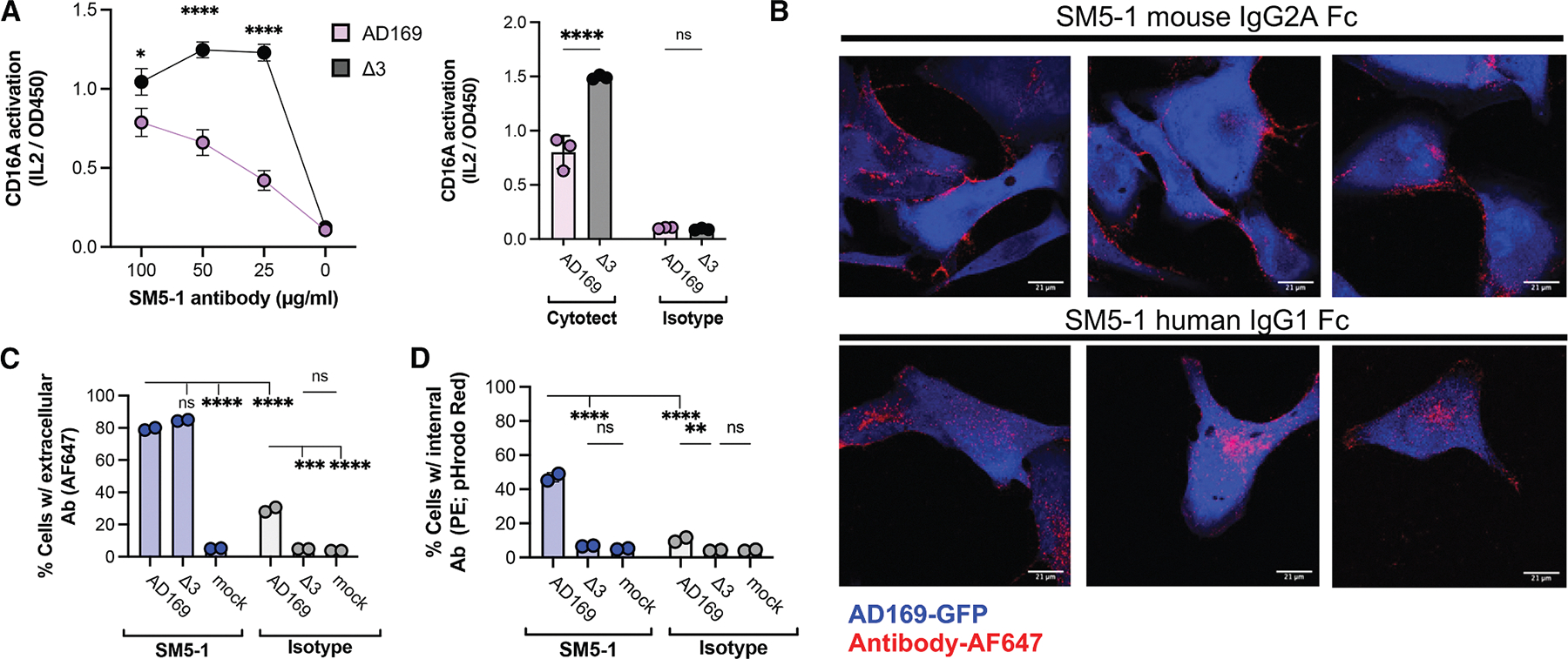
HCMV-infected cells capture human IgG1 antibodies to antagonize CD16A (A) BW-CD16A-ζ reporter cells were incubated with SM5–1 antibody, 100 μg/mL isotype, or 1:100 dilution Cytotect and AD169- or Δ3-infected MRC5 fibroblasts (MOI = 5 and 72 hpi), with CD16A activation quantified by mouse IL-2. (B) Fluorescent images of AD169-infected MRC5 cells (MOI = 2 and 96 hpi) incubated with AF647-labeled SM5–1 or SM5–1-mFc (20 μg/mL) for 2 h at 37°C. Cells fixed with 4% paraformaldehyde and scanned using Zeiss LSM 710/Elyra S.1 at 63×. Scale bars, 21 μm. (C and D) Flow cytometry measured the percent of cells positive for (C) extracellular or (D) internalized antibody after incubating 67 nM antibody with infected cells (as above) or mock-infected cells for 2 h at 37°C. Extracellular antibody detected with goat anti-human-Fcy AF647; intracellular with pHrodo-Red-labeled primary antibody. Data are mean ± SD, for *n* = 2 with *p < 0.05, **p < 0.01, ***p < 0.001, ****p < 0.0001, and ns: non-significant as determined by two-way ANOVA followed by Tukey’s multiple comparisons test in GraphPad. Data are representative of one experiment; each experiment was repeated twice.

We observed similar levels of SM5–1-mFc binding to MRC5 fibroblast cells infected with AD169 or Δ3 using flow cytometry and western blot, indicating similar gB expression levels (Figures S1A and S1B). Detection of the immediate-early antigen 1 and human leukocyte antigen (HLA) expression level by western blot confirmed the strains’ similar behavior.^31^ Infected cells were opsonized with antibody and incubated with a mouse BW5147-derived reporter cell to directly monitor the activation of human CD16A by mouse IL-2 secretion.^33^ We observed significantly greater activation when Cytotect or SM5–1 was incubated with Δ3 versus AD169-infected cells (*p* < 0.0001, Figure 1A), at 72 hpi (Figures S1C and S1D). Overall, vFcγR expression dramatically suppresses CD16A activation by SM5–1, which supports the use of this antibody in experiments to further define the contributions of these viral proteins to immune evasion.

Prior work suggested that gp34 and gp68 on the infected cell surface antagonize CD16A and frustrate NK cell activation by co-operating to bind human Fc domains and internalize the antibody/antigen complex.^23^ To monitor antibody binding and internalization into HCMV-infected cells, we combined fluorescently labeled antibodies with HCMV-infected fibroblasts in a series of experiments. Confocal imaging localized SM5–1-mFc to the cell periphery, consistent with Fab binding to cell-surface gB, while human SM5–1 was primarily internalized (Figure 1B). Flow cytometry analysis of this experiment performed on ice to prevent endocytosis showed that SM5–1 bound to AD169- and Δ3-infected cells similarly (~80% cells), while an isotype control preferentially bound to AD169-infected cells (~30% versus ~5% of uninfected cells; *p* < 0.001; Figure 1C), as expected for vFcγR capture of human Fc. When performed at 37°C, SM5–1 and the isotype control were internalized more by AD169 versus Δ3-infected cells (~50% versus 7%, respectively, for SM5–1, *p* < 0.0001 compared to ~10% versus 4% for the isotype, *p* < 0.01; Figure 1D). The increased antibody binding and internalization by AD169-infected cells support the concept of “bipolar antibody bridging,” in which the antibody Fab and Fc can simultaneously engage gB and vFcγRs on the infected cell surface,^34^ and led us to hypothesize that disrupting Fc:vFcγR interactions could increase antibody potency.

### Soluble, engineered gp34 and gp68 retain Fc-binding activities

We engineered the gp34 and gp68 ectodomains for soluble expression and used these proteins to roughly map the binding epitopes on human Fc and determine their binding kinetics. The truncated gp68 ectodomain (residues 69–289) was cloned and produced in CHO cells as previously reported,^35^ resulting in pure and homogeneous protein (t-gp68, Figure S2A). The gp34 ectodomain (residues 24–182) was produced similarly but displayed high levels of aggregation on non-reducing SDS-PAGE. Under reducing conditions, gp34 ran primarily as a single band at the expected size of ~35 kDa, suggesting cysteine scrambling complicated protein production (Figures S2B–S2D). Systematic substitution of the five cysteines with serine identified gp-34M with one C150S substitution, which exhibited reduced aggregation by size exclusion chromatography (SEC) and SDS-PAGE (Figures S2C and S2D). Notably, gp34-M measures ~35 kDa on SDS-PAGE, while SEC and static light scattering measurements indicate that it forms a ~75 kDa non-covalent dimer (Figure S2E). Gp34-M exhibited slightly enhanced human IgG1 binding versus unmodified gp34 by ELISA (Figure S2F), consistent with higher quality protein. These soluble proteins capture key characteristics of native gp34 and gp68 and can serve as tools to better understand Fc-vFcγR interactions.

### Human and viral FcγRs share overlapping but distinct epitopes on human IgG1

**Figure 2.**
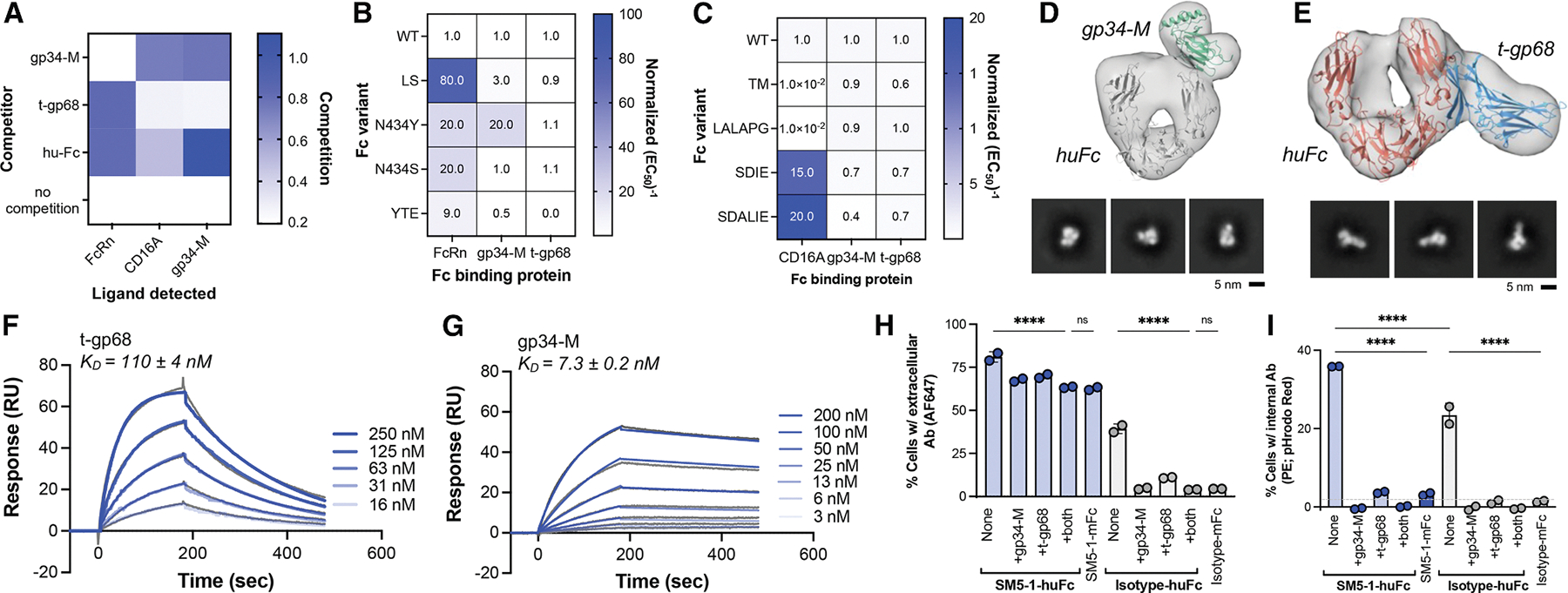
Fc binding to host Fc receptors inhibited by gp34-M and t-gp68 (A) Binding of host Fc receptors (FLAG-tagged CD16A-GST, FcRn-GST, or gp34-M) to immobilized human Fc was measured in the presence of competitor (gp34-M, t-gp68, and hu-Fc) by ELISA. Assay area under the curve (AUC) was normalized to controls lacking a competitor and presented as a heatmap. (B and C) Binding of antibody Fc variants to gp34-M, t-gp68, and (B) FcRn or (C) CD16A assessed by ELISA with normalized inverse 50% effective concentration (EC_50_) values shown as a heatmap with more color indicating better binding. (D and E) The nsEM 3D reconstruction and 2D class averages for particles containing (D) one Fc with one gp34-M bound near the CH_2_ tip and (E) one Fc with one t-gp68 bound to the CH_2_-CH_3_ interface. RoseTTAFold-based gp34 (green) and t-gp68 (blue) models fit the 3D reconstruction with the Fc crystal structure (PDB: 2GJ7). Scale bars, 5 nm. (F and G) SPR measured (F) t-gp68 binding to immobilized Fc and (G) binding of human IgG1 antibody to immobilized gp34-M with K_D_ values determined using BIAevaluation X100 software. (H and I) Flow cytometry measured (H) binding to and (I) internalization of pHrodo-Red-labeled antibody (67 nM) by AD169-infected MRC5 cells (MOI = 2 and 96 hpi) in the presence of t-gp68 (2 μM) and/or gp34-M (0.1 μM). Extracellular antibody detected with goat-anti-human-Fcy AF647; in (I), the dashed line represents the threshold for detection. Data shown are mean ± SD, *n* = 2, with *p < 0.05, **p < 0.01, ***p < 0.001, ****p < 0.0001, and ns, non-significant. All analyses are performed in GraphPad using two-way ANOVA with Tukey’s multiple comparisons test. Representative data of one experiment is shown; each experiment is repeated at least twice.

We mapped the vFcγR binding sites on Fc using gp34-M and t-gp68 in a series of ELISAs. These revealed that (1) the gp34 binding site overlaps with that of CD16A, (2) the gp68 binding site overlaps with FcRn, and (3) gp34 and gp68 can simultaneously bind Fc (Figure 2A). Fc variants with improved FcRn affinities revealed that the YTE (M252Y, S254T, and T256E) but not the LS (M428L/N434S) substitutions eliminated t-gp68 binding, implicating the YTE residues in the gp68 binding site (Figures 2B and S2G). Fc variants that do not bind CD16A largely retained t-gp68 and gp34-M binding, eliminating LALAPG (L234A, L235A, and P329G) and TM (L234F/L235E/P331S)^36,37^ as potential vFcγR contact residues (Figures 2C and S2H). Although these host and viral receptors bind overlapping sites on Fc, they appear to engage at least some unique hot spot residues, suggesting that Fc variants with impaired vFcγR binding that retain host Fc receptor binding could be identified.

As a complementary approach, we used negative-stain electron microscopy (nsEM) to visualize the gp34 and gp68 binding sites on human Fc. Purified Fc, gp34-M, and t-gp68 were combined in various ratios, and the complexes were purified by SEC before nsEM analysis (Figure S2I). Particles comprising only Fc and gp34-M suggest that one gp34 dimer binds one Fc on the upper C_H_2 region (Figure 2D), near the CD16A binding site. Additional particles with all three proteins or just Fc and gp68 (Figures 2E and S2I–S2K) suggest that t-gp68 binds the C_H_2-C_H_3 interface near the FcRn binding site and that two gp68 molecules can simultaneously bind one Fc. Our ELISA and nsEM data support a model proposed by Kolb et al.^23^ in which gp34 has a similar Fc-binding footprint as CD16A, while gp68 overlaps with the FcRn footprint.^35^

Finally, Fc-vFcγR binding kinetics were measured using surface plasmon resonance (SPR) (Table 1). Immobilized Fc exposed to varying t-gp68 concentrations yielded a K_D_ value of 110 ± 4 nM (Figure 2F), similar to previously reported values.^35^ Immobilized gp34-M followed by Fc injection yielded a K_D_ value of 7.3 ± 0.2 nM (Figure 2G). For both vFcγRs, the on-rates were modest (~5 × 10^4^ M^−1^s^−1^) as expected for proteins that colocalize on the cell surface, with intermediate (0.004 ± 0.001 s^−1^; t-gp68) or slow off-rates (0.0004 ± 0.0001 s^−1^; gp34-M). These binding kinetics are stronger than Fc interactions with CD16A and FcRn (~300–1000 nM depending on the allotype and 500 nM at pH 5.8, respectively^38^), indicating that vFcγRs can preferentially capture Fc.

### Soluble gp34 and gp68 inhibit internalization of anti-gB antibodies by HCMV-infected cells

To further define vFcγR roles in antibody internalization, we used soluble gp34-M and t-gp68 at a concentration of ~10 × K_D_ to competitively inhibit antibody capture by AD169-infected cells. When performed on ice to inhibit internalization, SM5–1 strongly bound AD169-infected fibroblasts (~75% positive cells), which was modestly reduced by the presence of one or both soluble vFcγRs (~60% positive cells, *p* < 0.0001; Figure 2H), as expected since gB binding would be unaffected. When incubated at 37°C, internalization of SM5–1 and the isotype control was high (35% and 25% positive cells, respectively) but greatly diminished by soluble vFcγRs (0–5% positive cells, *p* < 0.0001; Figure 2I). Notably, while we used soluble gp34-M in this assay, gp34, RL12, and RL13 are all members of the *RL11* gene family and likely share the same binding site on Fc; accordingly, soluble gp34 may block Fc capture by all three vFcγRs. These data indicate that Fc capture is selective for antigen-bound antibodies and can be completely blocked by soluble gp34 and gp68.

### Engineered Fc domains resist vFcγR capture

**Figure 3.**
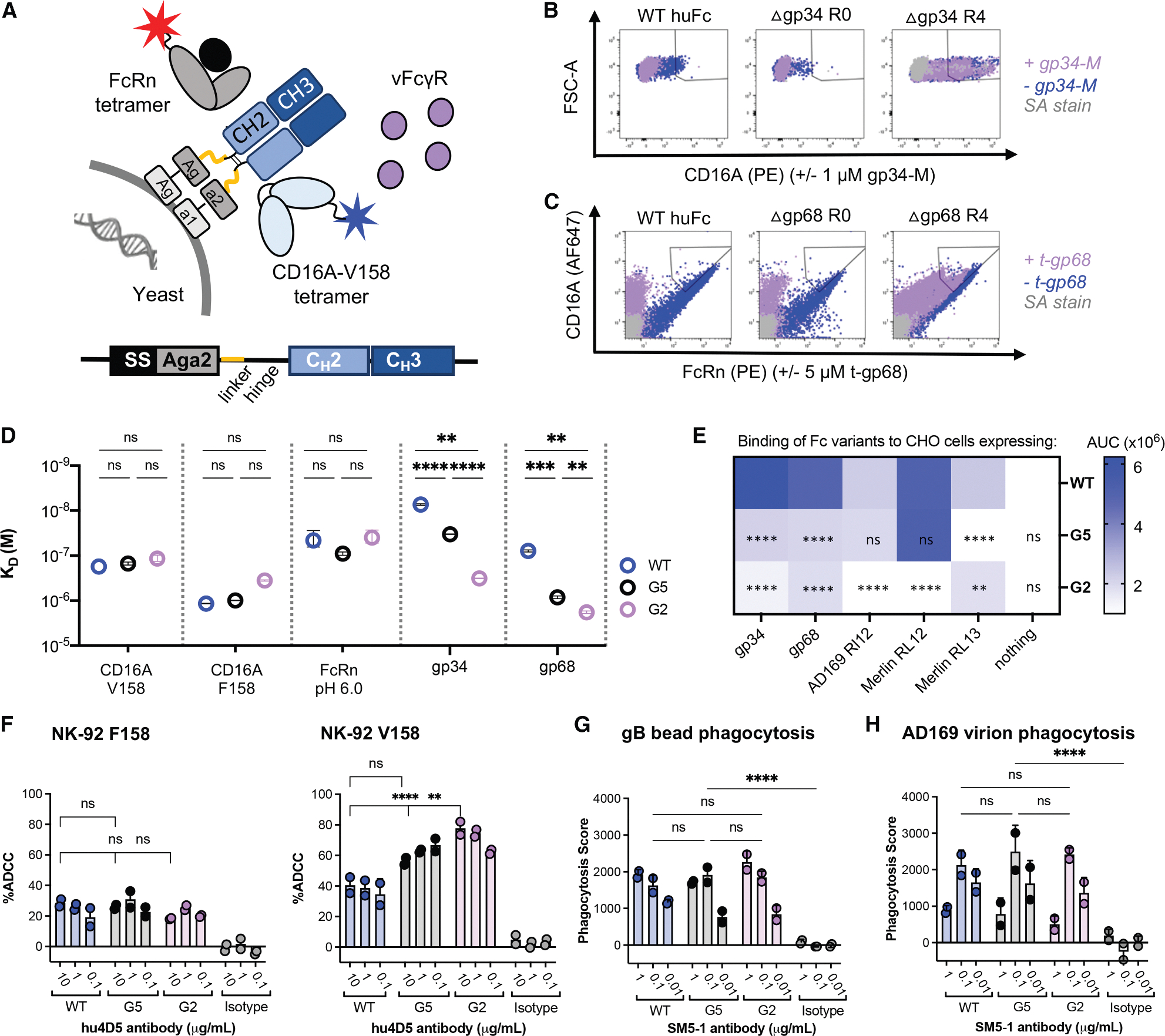
Engineered Fc domains resist vFcγR capture (A) Schematic of the yeast display and competitive staining strategy used for Fc selection. (B) To select for gp34-resistance, an Fc library (Δgp34 R0) was incubated with AF647-CD16A (V158) tetramers with 1 μM unlabeled gp34-M, followed by four rounds of FACS (Δgp34 R4). (C) To select for clones that also exhibit gp68-resistance, a library (Δgp68 R0) was constructed from R47 and incubated with AF647-CD16A (V158) and PE-FcRn tetramers at pH 6.0 with 5 μM unlabeled t-gp68, followed by four rounds of FACS (Δgp68 R4). (D) Binding affinities of Fc variant hu4D5 antibodies determined by BLI for FcRn, CD16A V158, or F158 or SPR for gp34-M and t-gp68, with the K_D_ mean ± SEM (*n* = 2) are shown. (E) Fc binding to different vFcγRs ectodomains expressed on CHO cells evaluated by staining with Fc variant hu4D5 antibodies and goat-anti-Fcγ-AF647 detection. Mean AUC shown as a heatmap, with darker color indicating more binding; repeated twice with *n* = 2. For (D) and (E) significance versus WT was assessed by one-way ANOVA with Tukey test for multiple comparisons. (F–H) ADCC was performed using hu4D5-Fc variant antibodies, HER2-positive SKOV3 target cells, and NK-92 V158 or NK-92 F158 effector cells, with cell lysis, was measured by calcein release. ADCP performed by incubating SM5–1 Fc variants with (G) gB-coated pHrodo-Green/APC-polystyrene beads or (H) pHrodogreen-labeled AD169 virions and then THP1 monocytes (50 beads or 2 PFU per cell). Phagocytosis scores were calculated as the percent antigen-presenting cell/fluorescein isothiocyanate (APC/FITC)-positive cells multiplied by the APC geometric mean fluorescence intensity (GMFI) and compared to WT. Representative data (mean ± SD) are shown, with each experiment repeated at least twice. Statistical analyses performed using two-way ANOVA followed by Tukey’s multiple comparison test, with *p < 0.05, **p < 0.01, ***p < 0.001, ****p < 0.0001, and ns, non-significant.

We engineered human IgG1 Fc variants with greatly reduced affinities for gp34 and gp68 while maintaining binding to CD16 and FcRn. This was achieved using a competitive yeast display strategy to select for variants that retained binding to host receptors FcRn and CD16A in the presence of an excess of unlabeled gp34-M and t-gp68. The human Fc residues 221–340, spanning the hinge, C_H_2 and C_H_3 domains, were fused to the C terminus of Aga2 and presented on the yeast surface in the same orientation as on an opsonized particle (Figure 3A). Flow cytometry showed that yeast expressing wild-type Fc bound fluorescent CD16A tetramers, this interaction was inhibited by soluble gp34-M (Figure 3B); Fc-expressing yeast also bound fluorescent FcRn tetramers at pH 6.0, this interaction was inhibited by soluble t-gp68 (Figure 3C), indicating that yeast-displayed Fc retains key biochemical features.

Sorting of a 1% error-prone library with >10^6^ variants for binding to CD16A-V158 tetramer (Figure 3B) yielded two Fc variants: one (S337F) with a single S337F change and another (R47) with four changes (H268L, E294K, Q311L, and K334E; Figure S3A). To identify a variant with reduced t-gp68 binding and potent CD16A recruitment, we amplified residues 221–340 of R47 under ~1% error-prone conditions, resulting in a second library with ~10^7^ variants. Sorting for binding to CD16A-V158 and FcRn tetramers at pH 6.0 (Figure 3C) yielded clone G2 with one additional consensus change: R255Q, which lies between the S254T and T256E residues of the YTE changes that also reduced gp68 binding (Figure 2B). We combined R255Q and S337F to generate variant G5; G5 and G2 also included a Y407V cloning artifact distant from the FcγR-binding regions. Notably, the S337F and R47 changes mediating gp34-resistance and the R255Q change conferring gp68-resistance occur near the CD16A and FcRn footprints, respectively, consistent with our epitope mapping (Figure 2A). These variants provide opportunities to evaluate the impact of Fc-resistance to a single vFcγR.

### Engineered Fc variants retain host Fc-receptor interactions

These five Fc variants (G2, G5, R255Q, R47, and S337F) and wild-type were each expressed with two Fab arms: the HER2-specific hu4D5 for Fc effector assays and the gB-specific neutralizing SM5–1 for anti-viral assays.^30^ Screening of SM5–1 Fc variants for binding to viral and host FcγRs by ELISA showed the greatest loss of vFcγR binding for G2 and G5, and these were selected as lead clones (Figures S3B–S3D). SPR showed a modest vFcγR affinity loss for G5 versus wild-type (K_D_ increased ~5-fold for gp34 and ~20-fold for gp68), while G2 exhibited dramatically reduced binding (K_D_ increased ~45-fold for gp34 and ~50-fold for t-gp68; Table 1; Figures 3D and S3E). Additionally, G2 and G5 maintained interactions with key host FcγRs, as measured by BLI (Table 2; Figure S3F), although slightly increased affinity for both CD16A allotypes was observed (Figure 3D).

To assess Fc binding on the cell surface, we expressed the vFcγR ectodomains with N-terminal FLAG tags on the CHO cell surface. As expected, G2 and G5 showed reduced binding to gp68 and gp34 compared to wild-type Fc (*p* < 0.0001; Figures 3E and S4A). Since the poorly conserved gp95 and gpRL13 are members of the *RL11* gene family with gp34, we speculated that gp34-resistant Fc variants may also lose binding to these proteins. Conserved homologies among these receptors^39^ allowed for identification of the putative immunoglobulin-fold domains for expression (Figure S4B). G2 exhibited reduced binding to gp95 alleles from the AD169 and Merlin strains (*p* < 0.0001), while G5 Fc had reduced binding to gpRL13 (*p* < 0.0001) compared to wild-type human Fc (Figures 3E and S4A). Fc binding to the Merlin gp95 and gpRL13 alleles was blocked by soluble gp34, suggesting that these *RL11* gene family members share overlapping antibody binding epitopes (Figure S4C). Interestingly, these Fc variants also resist capture by the HSV-encoded vFcγR homologs, gE/gI compared to wild-type Fc, when expressed on CHO cells (Figure S4D). Overall, we generated a panel of Fc domains ranging in affinity and specificity for multiple vFcγRs expressed by two herpesviruses.

### Engineered Fc variants retain key Fc-effector functions

We first evaluated antibody ADCC using hu4D5-containing antibodies, HER2-expressing SKOV3 target cells, and GFP+ human NK-92 effector cells^40^ expressing CD16A variants V158 or F158. For all Fc variants, the NK-92 effector cells expressing the low-affinity F158 CD16a-allele resulted in ~20%–30% target cell lysis. However, G2 and G5 showed increased target cell lysis versus wild-type Fc in the presence of NK-92 cells expressing the high-affinity CD16A-V158 allele (60%–80% versus ~30%–40% respectively, *p* < 0.001; Figure 3F). Phagocytosis has also been associated with protection from HCMV. Accordingly, ADCP was evaluated using human THP-1 monocytes (CD64^+^/CD32A^+^/CD16A^−^).^41^ All Fc variants exhibited similar phagocytosis scores for gB-coated beads or AD169 virions (Figures 3G and 3H). These engineered Fcs provide an opportunity to explore the impact of high, medium (G5), and low affinity (G2) capture by vFcγRs on Fc-dependent effector functions associated with HCMV protection.

### vFcγR-resistant Fcs mediate potent anti-viral responses against AD169-infected cells

**Figure 4.**
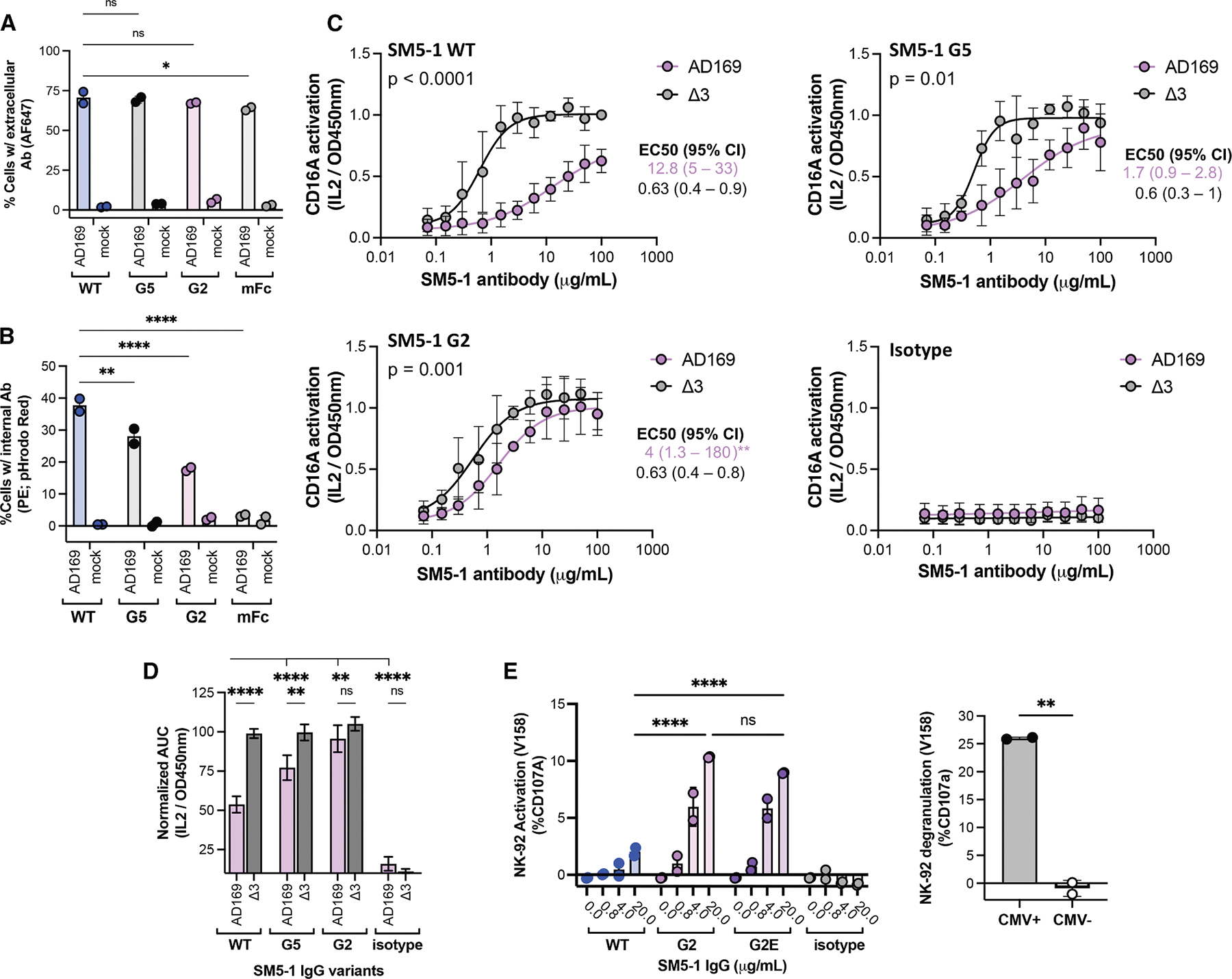
Engineered Fc domains enhance SM5–1 CD16A and NK activation against AD169-infected cells (A and B) (A) Cell staining by and (B) internalization of Fc variant SM5–1 antibodies (20 μg/mL) with AD169- or mock-infected MRC5 cells. Comparisons performed using one-way ANOVA with Tukey’s multiple comparisons; data are presented as mean ± SD (*n* = 2). (C) CD16A activation measured as mouse IL-2 production after incubation of SM5–1 antibodies with BW-CD16A-ζ reporter cells and AD169-or Δ3-infected MRC5 cells (MOI = 5 and 72 hpi). Data normalized to results for SM5–1 WT with Δ3; curves from *n* = 3 replicates were fit to 4PL or AUC using GraphPad. Mean EC_50_ values listed unless no activation was detected at 100 μg/mL; *p* values depicting EC_50_ differences for AD169 versus Δ3; * represents significance versus SM5–1 WT. (D) CD16A activation AUC normalized to SM5–1 WT with Δ3; data shown are the mean ± SEM of three experiments. (E) NK cell activation measured as percent CD107a-positive NK-92 (V158) cells after incubation with AD169-infected MRC5 cells (MOI = 2 and 96 hpi) and Fc-variant SM5–1 antibodies or polyclonal antibodies from Cytogam (CMV+) or CMV-negative serum (CMV−; 30 μg/mL). Data shown are mean ± SD (*n* = 2) and average of *n* = 3 experiments, with comparison using two-way ANOVA with Tukey’s multiple comparisons test and *p < 0.05, **p < 0.01, ***p < 0.001, ****p < 0.0001, and ns, non-significant.

To determine the impact of Fc engineering on anti-HCMV activities, we compared SM5–1 antibodies bearing different Fcs in a series of assays with AD169-infected cells. After staining on ice, extracellular antibody binding was similar for all variants due to Fab/gB interactions (~70%, Figure 4A). However, when incubated at 37°C, antibodies with wild-type Fc were efficiently internalized (~35%, similar to Figure 1C), which was reduced for G5 (25%) and further reduced for G2 (18%), with no internalization of the SM5–1-mFc control observed (*p* < 0.010; Figure 4B). To determine whether vFcγR-resistant Fcs improve NK cell activation, we monitored CD16A signaling by BW-CD16A-ζ reporter cells. While all SM5–1 antibodies demonstrated similar levels of potent CD16A activation against Δ3-infected cells, this was greatly suppressed when wild-type Fc was incubated with AD169-infected cells (12.8 and 0.63 μg/mL EC_50_ for Δ3 versus AD169, respectively; *p* < 0.0001). This difference was diminished for SM5–1 with G5 (4.0 versus 0.6 μg/mL EC_50_; *p* < 0.001) and further reduced for SM5–1 with G2 (1.7 and 0.6 μg/mL EC_50_; *p* < 0.01; Figure 4C). The assay area under the curve (AUC) summarizes these results, showing a large activation difference between Δ3 and AD169 for wild-type Fc, which is insignificant for G2 (Figure 4D). Analysis of additional Fc variants suggests that reduced binding to both gp34 and gp68 is required for CD16A activation, as neither R47 (gp34-resistant) nor R255Q (gp68-resistant) recapitulated G2 (Figures S5A–S5C). While G2 and G5 conferred slightly enhanced ADCC with NK-92 cells expressing CD16 V158 (Figure 3D), no improved binding to CD16A V158 was detected in binding assays or immune complex activation assays (Figures S5D and S5E), indicating that increased CD16A activation observed here is primarily due to reduced vFcγR capture.

During these studies, we noticed that Fc variant G2 had compromised developability characteristics, specifically rapid serum clearance from mice expressing human FcRn^42^ (Figure S6). Since biochemical binding to FcRn was not impacted (Table 2), we evaluated antibody maternal-fetal exchange, which is also driven by FcRn-mediated transcytosis^43^ but saw no significant difference for wild-type Fc versus G2 (Figure S6B). Suspecting that G2 stability was compromised, we individually reverted each change. This revealed that the E294K and Y407V reversions had minimal impact on binding to host or viral receptors but enhanced expression (Figure S6C). We created the final variant G2E, retaining the H268L, R255Q, Q311L, and K334E changes from G2 plus S337F to further reduce gp34 and gp68 binding. G2E exhibited similar vFcγR and host FcγR affinities (Figure S6C) but increased thermal stability, resistance to heat-induced aggregation (Figures S6D and S6E), and restored antibody pharmacokinetics relative to G2 (Figure S6A; Table S1). In addition, G2E mediated similarly high NK degranulation rates against HCMV-infected cells as G2 (up to ~14%, *p* < 0.0001), which compares well to CMV+ Cytogam (~25%), a high-titer human polyclonal antibody preparation binding many HCMV targets (Figure 4E). Collectively, these experiments show a correlation between reduced vFcγR-capture and enhanced CD16A activation, with G2 and G2E mediating potent CD16A activation against HCMV-infected cells.

### vFcγR-resistant Fcs mediate enhanced anti-viral activities against additional HCMV strains

**Figure 5.**
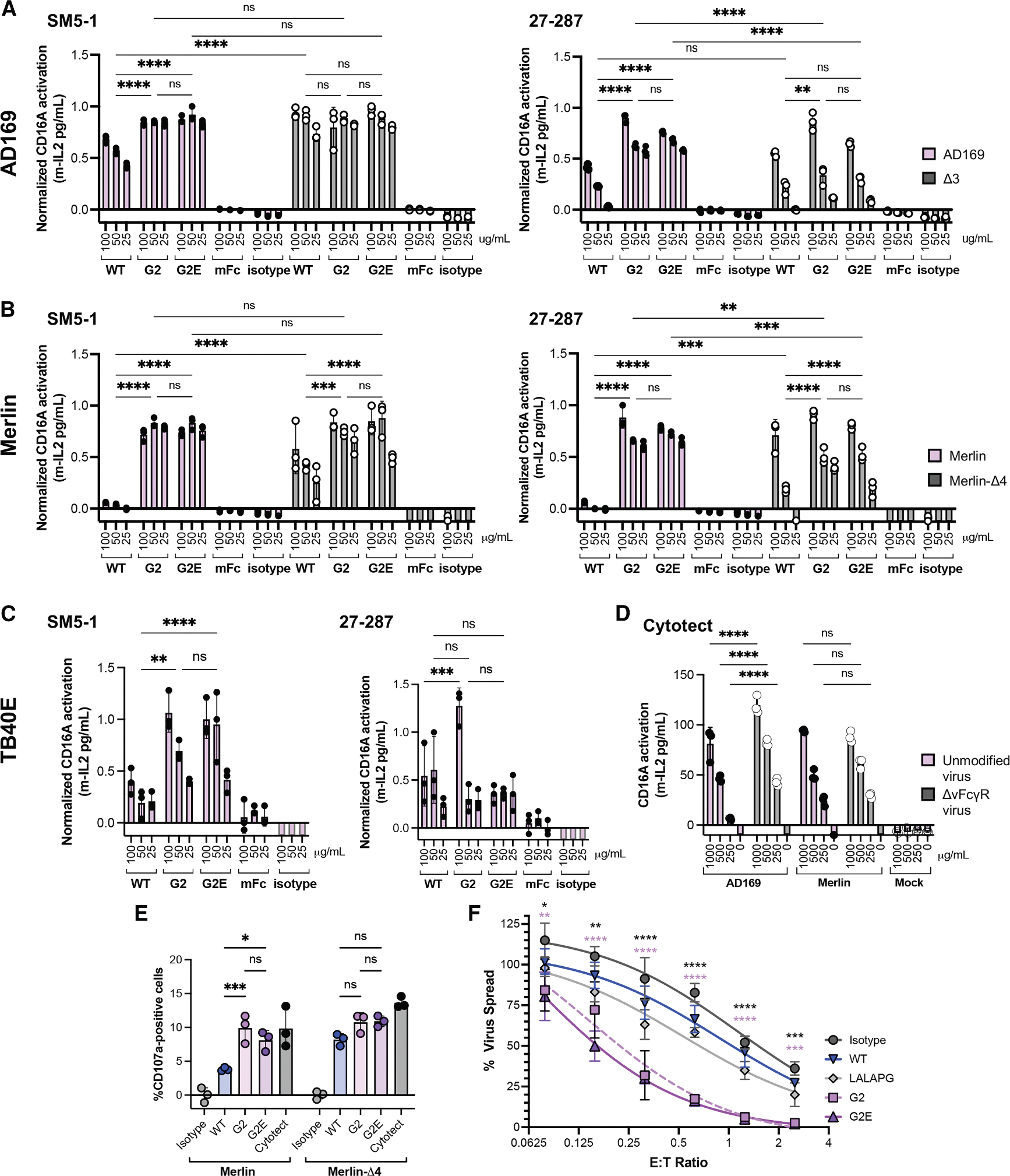
Engineered Fc domains mediate enhanced anti-viral activities against additional HCMV strains (A–C) CD16A activation measured as mouse IL-2 production after incubation of neutralizing SM5–1 or non-neutralizing antibodies with BW-CD16A-ζ reporter cells in the presence of HFF cells infected (MOI = 3 and 96 hpi) with (A) AD169 or Δ3, (B) Merlin or its vFcγR-deficient strain Merlin-Δ4, and (C) TB40E. (D) CD16A activation after incubation of Cytotect (500 μg/mL) with AD169, Merlin, or mock-infected cells. Data normalized to the maximum response per viral strain and presented as mean ± SD (*n* = 3) for one representative experiment, with significance determined by one-way ANOVA with Tukey test for multiple comparisons. (E) *Ex vivo* purified human donor PBMC cells and 27–287 antibodies (20 μg/mL) combined with Merlin- or Merlin-Δ4-infected HFF cells (10:1 E:T ratio or 1:1 NK:T ratio, 96 h hpi). NK cell degranulation (CD107a-positive) measured after 4 h; data shown are mean ± SD (*n* = 3) of one representative experiment with isotype control subtracted. (F) Immortalized skin fibroblasts (SFi-hTert) infected at low MOI, with 27–287 antibodies (5 μg/mL) and autologous NK cells added at indicated E:T ratios 72 hpi. After 7 days of culture in an Incucyte, the integrated GFP fluorescence was measured per antibody with NK cells relative to no NK cells. Each condition was assessed in technical quadruplicate, with curves fit to 4PL using GraphPad. Statistics compare G2 (black stars) or G2E (pink stars) to WT over different E/T ratios. Two-way ANOVA with Tukey’s multiple comparisons test was performed on all datasets in (A)–(E), except (C), with *p < 0.05, **p < 0.01, ***p < 0.001, ****p < 0.0001, and ns, non-significant.

To determine whether the beneficial effects of vFcγR-resistant Fcs can be generalized, we evaluated CD16A activation with another antibody and additional HCMV strains. The non-neutralizing antibody 27–287 binds antigenic domain 1 (AD-1) of gB^44^ and stains AD169-infected HFF cells less strongly than the AD-4 binding antibody SM5–1 (Figures S7A and S7B). Activation by 27–287 with Δ3-infected cells is similar but reduced compared to SM5–1 for all Fcs (Figure S7C), perhaps due to lower overall 27–287 binding. Similar to SM5–1, 27–287 mediated negligible NK cell degranulation when combined with a wild-type Fc and AD169-infected cells, which increased to ~8% when combined with the engineered Fc domains G2 or G2E (*p* < 0.0001 at 50 μg/mL; Figures 5A and S7C) despite similar antibody binding to infected cells (Figure S7D). This demonstrates that our engineered Fcs can enhance CD16A activation against AD169-infected fibroblasts for multiple gB antibodies.

We also observed enhanced CD16A activation using the clinical-like Merlin strain, which expresses all four vFcγRs, and the endotheliotrophic strain TB40E, which expresses the pentameric receptor-binding complex and three vFcγRs (gp34, gp68, and RL12). When combined with Merlin-infected cells, both SM5–1 and 27–287 showed strong binding to cells and CD16A activation with engineered Fcs, despite no activation by the wild-type Fc (Figures 5B and S7E). When a Merlin strain lacking all four vFcγRs (Merlin-Δ4) was used, both antibodies with wild-type Fc activated CD16A, which was enhanced by the engineered Fcs (*p* < 0.001 for SM5–1 and *p* < 0.0001 for 27–287 at 50 μg/mL). When combined with TB40E-infected HFF cells, both antibodies with G2 or G2E showed enhanced activation versus wild-type, despite low antibody binding (Figures 5C and S7F). The polyclonal control Cytotect showed strong CD16A activation against both AD169 and Merlin strains (Figure 5D). Finally, we observed enhanced degranulation using primary human-derived NK cells, SM5–1 antibodies, and Merlin-infected HFF fibroblasts. SM5–1 with G2 or G2E triggered comparable degranulation as Cytotect, which was all superior to wild-type Fc (Figure 5E). Across three HCMV strains and two antibodies, our engineered Fcs consistently improved CD16A and NK cell activation relative to wild-type Fc.

Finally, we used the non-neutralizing anti-gB antibody 27–287 in a virus dissemination assay to assess control of cell-to-cell spread by the Merlin strain expressing the full repertoire of immune-evasins. This provides a holistic readout for long-term virus control mediated by multiple mechanisms following low-level infection in the presence of NK cells and antibody.^32,45,46^ To ensure appropriate major histocompatibility complex:killer cell immunoglobulin-like receptor engagement, we used primary human donor NK cells with autologous skin fibroblasts. The isotype control antibody and 27–287 with wild-type or silent Fc showed similar results, while antibodies with engineered Fcs enhanced NK-mediated virus control at all cell ratios tested (*p* < 0.001; Figure 5F). This supports the potential for antibodies with engineered Fcs to enhance HCMV control against clinical strains expressing four vFcγRs and other NK cell-antagonizing proteins.

## DISCUSSION

Antibody therapeutics to treat HCMV have focused on neutralizing antibodies targeting gB or gH/gL-containing complexes that block viral entry. While these antibodies can prevent an initial infection,^47^ once established, HCMV is primarily cell-associated and spreads from cell-to-cell even in the presence of neutralizing IgG.^11,32,48–52^ This has prompted interest in understanding the role of Fc-mediated effector functions to enhance immune control of infection upon reactivation or reinfection. Fc modifications that enhance CD16A engagement increase ADCC against Ebola, HIV, and HCMV.^51,53–55^ These findings, coupled with reports that HCMV vFcγRs antagonize signaling through host FcγRs, led us to hypothesize that engineering antibody IgG1 Fc domains to resist capture by vFcγRs while preserving host FcγR binding could improve immune cell activation against HCMV-infected cells and confer enhanced viral control.

Protein engineering was central to achieving this goal. The conserved vFcγRs gp34 and gp68 both required modification (cysteine replacement and truncation, respectively^35^) to yield soluble constructs suitable for binding assays. Because we were unable to obtain high-resolution experimental or computational structures of Fc-FcγR complexes, we screened random mutagenesis libraries using yeast surface display with a competitive binding strategy to isolate clones binding FcRn and CD16A in the presence of unlabeled vFcγR. Interestingly, we identified an R255Q substitution, located between M252Y and T256E of the YTE half-life extension motif. While YTE reduced gp68 binding, it also compromised CD16A affinity and ADCC^56^; in contrast, R255Q reduced gp68 binding without impairing these functions. We also identified K334E and H268L changes near the Fc-CD16A interface, and Q311L, which was also found in the DHS extended half-life variant.^38^ Beyond HCMV, Fc engineering may be broadly applicable to antibodies targeting other pathogens expressing Fc-binding proteins, including other herpesviruses and bacteria like *Staphylococcus aureus*.^57^

When combined with engineered Fc domains, Fabs binding two different gB epitopes mediated strong CD16A signaling and NK cell degranulation against cells infected with three different HCMV strains. This dramatically contrasts with the negligible activation observed for these Fabs with wild-type Fc, seen here and previously.^12^ Enhanced CD16A activation can be attributed to vFcγR-resistance, as wild-type and engineered Fcs have similar activity against intact and isogenic viruses lacking vFcγR expression. In addition, antibody potency also appears to be influenced by the gB epitope. Neutralizing antibody SM5–1 binds an epitope present on pre- and post-fusion gB (PDB: 7KDD); epitope abundance likely supports the Fc clustering required for CD16A activation, resulting in strong SM5–1 responses. The non-neutralizing antibody 27–287, which binds an AD1 epitope that may only be accessible in post-fusion gB,^58^ also exhibited improved activity with engineered Fcs, although this is consistently reduced relative to SM5–1. Because gB is predominantly in the pre-fusion conformation,^59^ low epitope availability may restrict effective Fc clustering and limit antibody activity. Nevertheless, engineered 27–287 limited Merlin dissemination in the presence of human donor NK cells over several days. This clinical-like HCMV strain expresses a broader repertoire of immune-evasins than AD169, which may reduce antibody activity when using *ex vivo* NK cells. Despite this, CD16A activation can overcome some immune-suppression strategies^60^ and the resulting interferon gamma secretion can suppress virion production and cell-to-cell spread,^61^ as observed here. Collectively, these data demonstrate that engineering to reduce vFcγR capture yields potent antibody Fc domains that can be combined with multiple Fab arms to enhance the NK cell control of HCMV infection.

To better understand the basis for this activity, we examined the contributions of individual vFcγRs to antibody capture and CD16A antagonism. HCMV is the only herpesvirus encoding more than one vFcγR, suggesting that these perform complementary functions.^17^ Prior work by Kolb et al. proposed a cooperative binding model in which an antibody binds a viral antigen like gB, which positions its Fc for capture by adjacent gp34 and gp68 proteins to suppress CD16A signaling and facilitate antibody internalization.^23^ Our data are consistent with this model: the 10-fold stronger affinity of gp34 than gp68 for Fc (~7 nM versus 70 nM K_D_, respectively) suggests that gp34 initiates capture, with gp68 mediating internalization. Moreover, since gp34 directly interferes with Fc-CD16A engagement, CD16A activation is sensitive to small changes in Fc/gp34 affinity. This is exemplified by the S337F variant, which exhibits a 5-fold reduced gp34 affinity but markedly increased CD16A signaling and NK cell degranulation relative to wild-type Fc. Engineered Fcs that resist capture by both gp34 and gp68 show the additive effects expected by the co-operative model: G5 with modestly reduced binding to both vFcγRs has greater activity than variants with a single S337F or R255Q change. Across all our variants, loss of gp34/gp68 binding correlates with improved activity, exemplified by G2, which has the weakest vFcγR binding but greatest CD16A signaling and NK cell degranulation. These data support the hypothesis that vFcγRs antagonize CD16A activation and NK cell degranulation, while our engineered Fc variants provide a toolset to further define the impact of individual vFcγRs on Fc-mediated immunity.

Reduced vFcγR binding largely accounts for restored antibody activity, but the engineered Fcs also outperformed their wild-type counterparts in cells infected with Merlin strains lacking vFcγRs. Biochemical binding assays measured slight increases in CD16A affinity (200 versus 140 nM K_D_ for wild-type Fc and G2, respectively), while degranulation and CD16A activation assays performed without vFcγRs showed slight increases in basal CD16A activation. Together, these data suggest that engineered Fcs may better engage CD16A, which could contribute to Fc potency, although this effect is much less than the dramatic increases observed with HCMV-infected cells. Interestingly, Vlahava et al. demonstrated that independently increasing Fc affinity for CD16A enhanced ADCC against HCMV-infected cells. Monoclonal antibodies binding UL16 and UL141, two viral proteins that are expressed early after infection and before vFcγR levels rise, mediated ADCC against Merlin-infected cells when the Fc domain was modified with S239D and I332E changes to increase CD16A affinity to 2 nM^62^ without impacting vFcγR capture. High-affinity Fc domains conferred small increases in NK cell degranulation for some monoclonal antibodies, but much larger increases for cocktails of five antibodies.^32^ We reasoned that Fc modifications to reduce vFcγR binding would offer a broader therapeutic window, especially for antibodies targeting gB whose expression is synchronized with vFcγRs. Future work will explore the combinations of residue changes conferring vFcγR-resistance and CD16A-enhanced binding to further increase antibody potency as well as the impact of vFcγR-resistant antibody cocktails.

Antibodies bearing vFcγR-resistant Fc domains may be relevant for HCMV immune control *in vivo*. While CD8^+^ T cells are key to controlling HCMV in healthy individuals, accumulating evidence suggests that antibody-dependent NK cell recruitment also contributes to antiviral immunity. HCMV infection is associated with a pronounced expansion of NKG2C^+^/CD57^+^ and FcεR1γ-adaptive NK cells, which mediate ADCC.^63^ A recent serological study reported that reduced risk of fetal HCMV transmission correlated with ADCC activity mediated by UL16-binding antibodies.^14^ Conversely, individuals with impaired NK immunity are particularly susceptible to HCMV disease.^64,65^ These data suggest that antibody-mediated NK cell activation is an important component of a protective immune response against HCMV, but identification of appropriate viral targets and translating these observations into an effective therapeutic has been challenging. For example, gB-specific antibodies in polyclonal sera can drive some ADCC responses,^51^ but anti-gB monoclonal antibodies mediate very weak ADCC and NK cell degranulation *in vitro*.^14,32,66^ Recent data from animal models implicate vFcγRs in undermining antibody-mediated protection. Otero et al. deleted the gp34, gp68, and RL12 homologs from an RhCMV strain; subsequent infection of rhesus macaques showed accelerated viral control during primary infection that coincided with increases in anti-HCMV antibodies for animals infected with the vFcγR-deletion but not unmodified virus.^26^ Fc-engineered anti-HCMV antibodies could benefit transplant patients with impaired T cell function or congenitally infected infants whose outcomes depend on rapid virus control, but future studies are needed to define the clinical utility and scope of this approach.

### Limitations of the study

Our study provides proof-of-concept for an Fc engineering approach to enhance antibody-mediated NK cell activation and limit HCMV dissemination, but several limitations should be acknowledged. First, evaluation of antibodies binding additional antigens/epitopes will be needed to determine the generalizability of this approach. Second, while we focused on vFcγR effects, multiple other HCMV gene products can modulate NK cell activities by secreting inhibitors^67^ and altering cell-surface levels of ligands for activating and inhibitory receptors^16,68^; immune evasion strategies that will not be impacted by our Fc engineering approach. Third, adoptive transfer of polyclonal HCMV-specific T cells is a promising approach to control infection in solid organ and stem cell transplant patients.^69^ A better understanding of the contributions of different immune effector cells to HCMV control (CD8^+^ T cells, NK cells, and macrophages) will help identify clinical situations appropriate for antibody versus T cell interventions. Finally, our conclusions are based on *in vitro* studies since the requirement for cognate Fc-host Fc receptor and Fc-vFcγR interactions, and divergence of viral homologues between species^70^ complicates the use of animal models. Future experiments using the *Rhesus macaque* model will be a key step in assessing the clinical relevance of this approach.

## RESOURCE AVAILABILITY

### Lead contact

Further information and requests for resources and reagents should be directed to and will be fulfilled by the lead contact, Jennifer A. Maynard (maynard@che.utexas.edu).

### Materials availability

Plasmids generated in this study will be made available on request by the lead contact with a completed materials transfer agreement.

### Data and code availability

- All data reported in this paper will be shared by the lead contact upon request.
- This paper does not report original code.
- Any additional information required to reanalyze the data reported in this paper is available from the lead contact upon request.

## STAR★METHODS

### EXPERIMENTAL MODEL AND STUDY PARTICIPANT DETAILS

#### Cell lines

The following cell lines were obtained from ATCC. CHO-K1 (CCL-61), MRC-5 fibroblasts (CCL-171), HFF fibroblasts (SCRC-1041), and SKOV3 cells (HTB-77) were maintained in complete medium (DMEM, 10% FBS, and 100 U/mL penicillin) at 37°C/5% CO_2_. The human monocytic line THP-1 (ATCC TIB-202) was maintained in RPMI media (10% FBS, 100U/mL penicillin) at 37°C/5% CO_2_ and NK-92 cells expressing the V158 and F158 CD16A alleles (ATCC PTA-8836 and 8837, respectively) were maintained in α-MEM media (10% FBS (Gibco), 10% horse serum (Thermo Fisher Scientific), 0.2 mM myo-inositol (Sigma, #I7508–50G), 0.1 mM β-mercaptoethanol (Sigma, #636869), 0.02 mM folic acid (Sigma, #F8758–5G), 1.5 g/L sodium bicarbonate, 1 mM non-essential amino acids (Thermo Fisher, #11–140-050), 1 mM sodium pyruvate (Gibco# 11360–039), 2 mM glutamine, supplemented with 200 rU/mL of IL-2 (Sigma, #SRP3085–50UG), at 37°C/5% CO_2_. ExpiCHO (A29133) and Expi293 cells (A41249) were purchased from Thermo Scientific and maintained in ExpiCHO expression medium and Expi293 expression medium at 37°C, 8% CO_2_ respectively. BW5147 mouse thymoma cells (kindly provided by Ofer Mandelboim, Hadassah Hospital, Jerusalem, Israel) were maintained in RPMI (10% FBS, 0.5% pen/strep (PAN Biotech #P06–07100) sodium pyruvate (1×, Gibco #11360–039) and β-mercaptoethanol (0.1 mM, Sigma #636869)) at 37°C/5% CO_2_. BeWo b30 cells (Accegen ABC-TC535S) were cultured in DMEM supplemented with 10% FBS in a 5% CO_2_ atmosphere incubator at 37°C. Human fetal foreskin fibroblasts (HFF) and local donor skin fibroblasts (SF) immortalized with human telomerase,^82^ or HFFF-hTert expressing the tetracycline repressor,^73^ were maintained in complete medium (DMEM, 10% FBS) at 37°C/5% CO_2_.

#### Animals

Transgenic homozygous Tg32 mice expressing human FcRn (The Jackson Laboratory Cat #014565) were used to determine antibody clearance rates *in vivo*. A total of 3–7 mice were used for studies with males and females. All animal experiments were performed in compliance with the US National Institutes of Health *Guide for the Care and Use of Laboratory Animals* under protocols AUP-2018–00093 and AUP-2022–00229 approved by the University of Texas Institutional Animal Care and Use Committee.

#### Virus stocks

The following stocks were produced and used throughout the studies: BAC2-AD169/GFP (gift from Professor Thomas Shenk, Princeton University); BAC2-AD169-varL; BAC2-AD169-varL ΔRL11ΔUL118–119 ΔRL12 (Δ3). Deletion virus mutants were generated as described previously (Kolb et al., 2021) using the primers listed in Table S1. In brief, recombinant HCMV AD169 mutants were generated according to previously published procedures^83,84^ using pAD169-BAC2 (MN900952.1,^85^) corresponding to AD169varL (Le et al., 2011) as the parental genome. For the construction of the HCMV deletion mutants, a PCR fragment was generated using the plasmid pSLFRTKn^20^ as the template DNA. The PCR fragment containing a kanamycin resistance gene was inserted into the parental BAC by homologous recombination in *E. coli*. The inserted cassette replaces the target sequence which was defined by flanking sequences in the primers. This cassette is flanked by *frt*-sites which can be used to remove the kanamycin resistance gene by *FLP*-mediated recombination. The removal of the cassette results in a single remaining *frt*-site. The deletion of multiple non-adjacent genes was conducted in consecutive steps. The gene *TRL11* was deleted by use of the primers KL-DeltaTRL11-Kana1 and KL-DeltaTRL11-Kana2 The gene TRL12 was deleted by use of the primers KL-DeltaTRL12-Kana1 and KL-DeltaTRL12-Kana2. The gene UL119 was deleted by use of the primers KL-DeltaUL119-Kana1 and KL-DeltaUL119-Kana2.

Merlin was derived from a BAC encoding the complete genome of strain Merlin, which matches the genome of the original clinical virus within a patient.^73^ Merlin Δ4 was constructed by deleting RL11, RL12, RL13, and UL119 by en-passant modification and has been described previously.^32^ Merlin-GFP was deleted for genes UL128 and RL13 to enable it to spread more rapidly in fibroblast cultures, and contains a P2A-GFP cassette after the UL36 gene.^74^ All Merlin viruses underwent whole-genome sequencing to verify genomic integrity following recovery from the BAC.^86^

#### HCMV infection

For staining and *in vitro* assay experiments, MRC-5 or HFF fibroblasts were cultured in complete medium (DMEM (Gibco), 10% Fetal Bovine Serum, 100 U/mL Penicillin) to 80% confluency in 96-well plates prior to infection with HCMV AD169-GFP, AD169-varL, AD169-Δ3 or TB40E^75^ at the MOI noted. Media was unchanged and virus was allowed to incubate 72–96 h prior to performance of experiments. The pp71 plasmid and AD169/GFP BAC was a gift from the Shenk Lab. Both plasmids were electroporated into 1E6 MRC-5 cells and allowed to recover to 2–3 weeks prior to subculturing AD169/GFP containing media onto larger confluent flasks of MRC-5 (15–20 flasks). For AD169-varL strains, 15–20 flasks of MRC5 were infected at an MOI of 0.1–0.02. All infected cells were incubated for 11–14 days at 37°C/5% CO_2_ prior to harvesting supernatant and concentrating virus in media using 20% sorbitol cushion. Pellets were resuspended in filter sterilized in 7% sucrose/1%BSA/PBS and aliquots were stored at −80°C. Concentration of stored virus is determined using plaque assay or limiting dilution method.

Merlin strains were grown in HFFF or HFFF-TetR as required.^73^ Flasks were infected at MOI = 0.03 and incubated until 100% CPE, at which point supernatant was harvested, cells removed by low-speed centrifugation, and virus pelleted by high-speed centrifugation before being resuspended in complete medium. Aliquots were stored at −80°C and titers determined by plaque assay. To infect HFFF-hTert or SF-hTert, cells were plated at 80% confluency in DMEM lacking FCS, then infected at MOI = 5 for 2h, after which cells were maintained in complete media.

### METHOD DETAILS

#### Preparation of recombinant proteins

UL118-UL119 (gp68) was acquired through Uniprot (P16809) and was codon optimized (IDT). RL11 (gp34, Uniprot 16809) was cloned from the AD169-GFP BAC plasmid (a gift from the Thomas Shenk Lab at Princeton University). Both receptors (gp68; 68–289 and gp34; 24–182) were cloned into the pcDNA3.0 vector with a c-term FLAG tag (M2) and single or twin strep tag. All DNA oligos were purchased from IDT and are listed in Table S1. CHO cells were then used to produce soluble gp34 constructs, cells were transfected in low-IgG medium (10% low IgG FBS + DMEM) using Lipofectamine 2000 (Thermo Fisher). Day 1 of transfection cells were incubated at 37°C/5% CO_2_ and after 24 h media was changed and cells were allowed to express protein at 32°C/5% CO_2_ for an additional 4 days. Media was then harvested and protein was isolated using Strep-tactin XT column (IBA) on AKTA Pure FPLC. Wash buffer consisted of 100 mM Tris-HCl, 150 mM NaCl, pH 8.0, and elution buffer was the same as wash buffer plus 50 mM biotin (Sigma). ExpiCHO high-yield cells were used to produce soluble t-gp68 constructs. Manufacturer’s transfection guidelines were used; however, cells were only allowed to express gp68 for maximum of 5 days at 32°C, 5% CO_2._ Proteins were purified via Streptactin XT column on AKTA Pure FPLC (GE Healthcare) according to the manufacturer’s instructions. All proteins were buffer exchanged into PBS using Amicon Ultra-30 centrifugal spin columns (Millipore), aliquoted and frozen down at −80°C.

Host Fc receptor plasmids CD16A V158, CD16A F158, FcRn-GST and CD16-GST were reported in^87^ and the gB post-fusion plasmid were previously reported in.^58^ We used these plasmids to transfect Expi293 and purified the individual receptors (CD16A V158, CD16A F158) using IMAC Sepharose resin (Cytiva). Wash buffer consisted of PBS and elution was performed using 100 mM EDTA+PBS. The gB post-fusion was purified using Streptactin XT on AKTA Pure FPLC (GE Healthcare) as previously described. The GST labeled receptors were purified using GSTrap columns (Cytiva) on the AKTA Pure FPLC (GE Healthcare). Elution was performed using 10mM reduced glutathione. All proteins were buffer exchanged into PBS using Amicon Ultra-10 centrifugal spin columns (Millipore).

#### Antibody expression and purification

Full-length antibody versions of all Fc variants were cloned as previously described^88^ with the following Fabs: hu4D5,^77^ SM5–1,^30^ mouse 27–287,^89^ and isotype control 2B1 which binds the *Bordetella pertussis* antigen pertactin).^90^ Antibodies were expressed in ExpiCHO (ThermoFisher Scientific) cells according to the high titer protocol and purified on a Protein A HiTrap column (GE Healthcare) with the AKTA Pure FPLC system (GE Healthcare). The column was washed using 25 mM Tris-HCl and 25mM NaCl, pH 7.2. Antibodies were eluted using 100mM sodium citrate at pH 3.0. All antibodies were then buffer exchanged into PBS using Amicon Ultra-30 centrifugal spin columns (Millipore). Each purified antibody was loaded at 3 μgs per well in 4–20% SDS-PAGE (Bio-Rad) gels under reducing and non-reducing conditions and analyzed by analytical size exclusion chromatography on a Superdex S200 column (Cytiva). The human Fab domains of SM5–1 and the isotype control 2B1 were cloned into the mouse IgG2A Fc domain in a pCDNA3.0 vector. Protein production and purification was performed as described^91^ using ExpiCHO cells and protein A chromatography.

#### Expression and activity of recombinant viral Fc receptors on CHO cell surface

Extracellular ectodomains of gp68 (Uniprot: P16739; aa# 26–289), gp34 (Uniprot Q6SWD1; aa# 24–182), Merlin RL13 ΔNT (Uniprot: Q6SWC9; aa# 100–246^92^), AD169 gp95 (Uniprot: B8YE37; aa# 32–369), Merlin gp95 (Uniprot: Q6SWD0; aa# 39–366) were cloned into the pcDNA3.0 vector with a murine IgK signal sequence, c-term FLAG (M2) tag, and the PDGFRα transmembrane domain using the primers in Table S1. Plasmids were transfected in 2 mLs of ExpiCHO cells using manufacturer’s protocol and were allowed to express for 48 h at 37°C/8% CO_2_ in a 6 well plate (Corning). Cells were washed with wash buffer (1% FBS + PBS) and seeded at 1E5 per well in a 96-well v-bottom plate (Corning) prior to staining. Serially diluted antibody variants (1000, 500, 250, 125, 62.5, 31.2, 15.6, 0 nM) were used to stain cells in duplicates on ice for one hour. Cells were then washed 3 times in wash buffer prior to addition of anti-FLAG (M2)-PE (Biolegend, 637309) and goat-*anti*-human Fcγ-AF647 (Jackson Immuno-Research, 109–606-170) secondaries at a 1:500 dilution. Staining was done on ice for one hour prior to washing three times and reading samples on the Fortessa using the HTS plate reader. The percent of positive cells for both PE and AF647 were then gated and the GMFI of this population was reported. The GMFI for control wells with no antibody added was subtracted from the experimental values.

#### Library construction, yeast display, and FACS screening

Human IgG1 (aa# 233–437) was cloned using specific primers (5′ hinge CH_2_ and 3′ CH_3_, see Table S2) into the pCTcon2^93^ vector at the c-terminus of Aga2 and transformed into the EBY100 (ATCC MYA-494,^94^ chemically competent cells using the Frozen-EZ Yeast Transformation II Kit (Zymo Research T2001). Transformations were selected based on the Trp auxotrophic marker on selective media plates (6.7 g/L Yeast Nitrogenous Base (YNB), 20 g/L casamino acids (CAA) 2% glucose, 100 U/mL pen-strep, 18 g/L agar) and allowed to grow at 30°C for 2 days. Yeast colonies were verified for sequencing using colony PCR followed by Sanger Sequencing. Library PCRs were first generated with shorter primers (5′ hinge CH_2_ and 3′ CH_2_) on the CH_2_ domain of the Fc sequence (residues #221–340). Error prone PCR was done using Taq DNA polymerase with different ratios of buffers and dNTPs.^95^ Longer primers (5′ hinge CH_2_ long and 3′ CH_2_ long) were used to amplify the PCR product and introduce at least 40 base pairs overhangs. The pcTCon2 vector was digested using *Xma*I (NEB) and *Pst*I (NEB). Fresh EBY100 electrocompetent cells were generated and 200 μLs was added to 2mm electroporation cuvettes mixed with 2 μgs of vector and 4 μgs of PCR insert. Five transformations were generated using Gene Pulser (BioRad) per library and allowed to recover in YPD medium prior to spinning down and resuspending in 100 mLs of selective medium (YNB, CAA, pen-strep, 2% glucose). Libraries were grown at 30°C overnight shaking at 250 rpm and were further passaged into induction medium (YNB, CAA, pen-strep, 2% galactose) at 20°C. Library size was determined by diluting recovered transformations onto selective media agar plates. A final error rate of 1% was generated for both libraries verified by colony PCR and Sanger Sequencing of 10 individual yeast colonies.

For staining the library, monomeric biotinylated CD16 V158 (Sino Biological) and biotinylated FcRn (Acro Biosystems) were made into tetramers using streptavidin-AF647 (Jackson ImmunoResearch, 016600084) or streptavidin-PE (BioLegend, 405204). To sorting for loss of gp34 binding, the library was passaged into selective media and then induced, as previously mentioned, prior to staining. For the first round of sorting, gp34 library (10^7^ cells) were centrifuged at 1000xg, 5min and stained in 1 mL of 10 nM of CD16 V158 tetramer (AF647) in sterile wash buffer (1% BSA + PBS) for one hour on ice. It was then washed 3× in wash buffer and sorting for CD16A binding clones (1% of population) was performed on the FACS FusionAria. At least 1×10^5^ cells were collected in selective media and allowed to grow up at 30°C before inducing. Further rounds of sorting included 10 nM CD16A V158 tetramer (AF647) in the presence of unlabeled 5 μM gp34-M, and at least 0.5–1% of the population was collected. Sorting in the presence of excess amounts of soluble gp34-M was performed three times in total.

To sort for loss of gp68 binding, the library was passaged in selective media and induced, as previously mentioned, prior to staining. For the first round of sorting, gp68 library (10^7^ cells) stained in 1 mL of 10 nM of CD16 V158 tetramer (AF647) and 10 nM of FcRn tetramer (PE) in sterile wash buffer at pH 6.0 (1% BSA + PBS) for one hour on ice. It was then washed 3× in wash buffer (pH 6.0) and sorting for CD16A-AF647/FcRn-PE binding clones (1% of population) was performed on the FACS Fusion Aria. At least 10^5^ cells were collected in selective media and allowed to grow up at 30°C before inducing. Further rounds of sorting included 10 nM CD16A V158 tetramer (AF647) and 10 nM of FcRn tetramer (PE) in the presence of unlabeled 10 μM t-gp68, and at least 0.5–1% of the population was collected. Sorting in the presence of soluble t-gp68 was performed 2 times in total. In the final round, 5 μM of gp34-M was added alongside t-gp68, FcRn, and CD16A to ensure no recovery of gp34 binding was introduced.

Rounds from libraries were plated on selective media plates and allowed to grow for 2 days in 30°C incubator with 10 colonies per round subjected to colony PCR. Fragments were purified using Zymo Gel and DNA Recovery Kit and submitted to Sanger Sequencing. Unique yeast clones were grown up, induced and characterized for binding to FcRn (pH 6.0), CD16A V158, gp34-M, and t-gp68 using BD Fortessa. Clones that maintained binding to FcRn (pH 6.0), CD16A, and lost binding to gp34-M (library 1) or t-gp68 and gp34-M (library 2) were then cloned into full length IgG1 antibodies.

#### Differential scanning fluorimetry

The thermal unfolding temperatures of the antibodies (0.250 mg/mL) were assessed in duplicate using the Protein Thermal Shift Dye Kit (Thermo Fisher Scientific) according to the kit instructions. Continuous fluorescence measurements ($\lambda_{\text{ex}}=580\;nm,\lambda_{\text{em}}=623\;nm$) were performed using a ThermoFisher ViiA 7 Real-Time PCR System, with a temperature ramp rate of 0.05°C/s increasing from 25°C to 99°C.

#### Enzyme-linked immunosorbent assay (ELISA)

Competition ELISA with recombinant protein that included FLAG tag (CD16A GST, FcRn GST, and gp34-M). Unlabeled competitors (gp34-M, t-gp68) were diluted down the plate by 5-fold and incubated with labeled proteins (2–5 μg/mL). Competition was analyzed as the knockdown of labeled protein using an anti-FLAG (M2) HRP antibody (Sigma A-8592). FcRn-GST binding was performed at pH 6.0, whereas the rest of the assays were performed in 5% milk PBST at pH 7.4. Two methods were performed for ELISAs, either receptor-coated or antibody-coated. For receptor binding ELISAs, soluble receptors were coated for one hour at room temperature at 4 μg/mL (t-gp68, gp34-M, CD16A-GST, or FcRn-GST (pH 6.0)). Human IgG1 antibodies (hu4D5 WT, LS, N434S, N434Y, and YTE) were diluted down 5-fold (starting at 25 μg/mL) in the presence of 5% milk PBST and binding was detected using an anti-κ HRP antibody (Southern Biotech 2060–05). For antibody binding ELISAs, antibodies (hu4D5 WT, S337F, R47, R255Q, G2, G5, YTE) were coated for one hour at room temperature at 4 μg/mL and then blocked with 5% milk PBST. Labeled receptors, t-gp68, gp34-M, CD16A-GST, or FcRn-GST (pH 6.0 or 7.4) were serially diluted from 450 nM with 5-fold dilution steps in the presence of 5% milk PBST and binding was detected using an anti-FLAG-M2-HRP antibody. All curves were fit with 4PL curve and the AUC calculated and compared using GraphPad.

#### ADCP of gB coated beads and labeled AD169 virions with THP-1

For bead phagocytosis, red fluorescent polystyrene beads (Bangs Laboratory, FSDG004) were washed with PBS three times in an Ultrafree Centrifugal Filter (0.22 mm, Millipore) and resuspended in PBS with 100 μg/mL purified gB protein. The beads were incubated in the dark for 1 h, before removing excess gB by filtration. Beads were then labeled with 1:100 pHrodo Green iFL STP ester (Thermo Fischer Scientific, P36013) in PBS-5% FBS, with excess pHrodo removed by filtration for a final concentration of 5×10^8^ beads/mL. For virion phagocytosis, approximately 3×10^6^ AD169 virions were thawed from −80°C, buffer exchanged in PBS, and concentrated to a final volume of 100 μL using filtration spin columns (Amicon Ultra-10, Millipore). Phrodo iFL Red STP Ester (5 μL, Thermo Scientific) was added for 1 h at room temperature before buffer exchanging with PBS to remove excess dye by filtration and diluted to 1×10^6^ PFU/mL in complete media (RPMI + 10% FBS).

To assess bead/virion phagocytosis, antibodies were serially diluted in 50 μL complete media (RPMI + 10% FBS) and combined with 50 ml beads/virions and THP-1 cells (25,000 cells/well in 100 μL complete media) in a 96-well U-bottom tissue culture treated plate (Corning). This resulted in final ratios of 50 beads or 2 PFU per cell. After 4 hours at 37°C/5% CO_2_, internalization was stopped by placing plates on ice and washing three times in cold PBS + 5% FBS before analysis using the PE, AF647, and FITC channels on the Fortessa HTS plate reader. For bead ADCP, phagocytosis was measured as the percent of bead-positive (AF647) cells divided by the percent of pHrodo-positive (FITC) cells, then multiplied by the GMFI of the AF647-positive population. For virion ADCP, phagocytosis was measured as the percent of pHrodo-positive (PE) cells multiplied by the GMFI of the PE-positive population. The average value for control wells lacking antibody was subtracted to give the final values reported.

#### ADCC assays with SKOV3

ADCC assays were performed with SKOV3 cells using the NK-92 (F158 or V158) cell lines. SKOV3 cells were labeled in serum-free medium with 2 mM calcein-AM (BD Pharmingen) for 30 min in the dark at 37°C/5% CO_2_. Calein-labeled SKOV3 cells were spun down and resuspended 3 times in complete media (DMEM +10% FBS). NK-92 cells were also spun down and resuspended in complete media at 2×10^6^ cells/mL. SKOV3 cells were seeded at 10,000 cells/well (100 μL of 10^6^ cells/mL) in a 96 U bottom tissue culture treated plate (Corning) and 50 μLs of antibody diluted in complete media was added (10, 1, and 0.1 μg/mL final concentrations). NK-92 cells (50 μL) were added for a final E:T ratio of 10:1. ADCC incubation was performed at 37°C/5% CO_2_ for 4 h. Cells were then spun down at 300 × g and 100 μLs of supernatant was transferred to clear-bottom black 96 well plate (Corning). Fluorescence (emission and excitation 480/525) was collected using a SpectraMax M5 plate reader. The percent ADCC was measured using the equation: (Experimental–SpontaneousRelease)*100/(Maximumlysis–Experimental). Maximum lysis is SKOV3 cells lysed with RIPA buffer (Thermo Scientific) and spontaneous release was measurement of SKOV3 cells with no antibody or NK-92 cells.

#### IgG transcytosis assay

BeWo b30 cells (AcceGen Biotech) were seeded at 12×1^4^ cells/cm^2^ in polyester Transwell permeable support inserts (0.4 μm pore size) and grown to a trans epithelial electrical resistance (TEER) of ~400 Ω (132 Ω/cm^2^). TEER is measured using an EVOM2 and STX chopsticks by WPI. After starvation for 1 h (DMEM w/o supplements), cells were cooled on ice, washed with cold HBSS pH 6 (top compartment) and 200 μL pre-cooled IgG solution (0.1 mg/mL in HBSS pH 6) added to the top compartment; the bottom compartment was filled with culture medium. Plates were moved to a 5% CO_2_ atmosphere incubator at 37°C. The antibody concentration of samples from the bottom compartment were quantified via commercial anti-human IgG ELISA kit (Abcam) for each time point.

#### BW-CD16A-ζ activation assays with HCMV-infected fibroblasts

This reporter assay is based on BW5147 reporter cells stably expressing human CD16A allele 158V or 158F (as indicated), as a chimeric molecule providing the ectodomain of the FcγR fused to the transmembrane domain and cytosolic tail of mouse CD3ζ.^33^ Briefly, MRC-5 or HFF fibroblasts were infected at MOI of 2–5 (as indicated) with various HCMV strains at specific time points. These were then used as target cells and pre-incubated with titrated amounts of antibody as indicated in medium (RPMI) supplemented with 10% FBS for 30 min at 37°C/5% CO_2_. After opsonization, target cells were washed with PBS + 10% FBS and co-cultured with BW5147-CD3ζ reporter cells (20:1 effector:target ratio) expressing the human CD16A ectodomain for 16 h at 37°C/5% CO_2_. Secretion of mIL-2 by the reporter cells was quantified by anti-mIL-2 sandwich ELISA as described previously.^33^

#### CD107a NK cell degranulation assay with HCMV infected cells

NK-92 V158 or F158 were resuspended at 2×10^6^ cells/mL in the presence of 6 μg/mL Golgi Stop (Monesin, BD Pharmingen) and 10 μg/mL Golgi Plug (Brefeldin A, BD Pharmigen) and anti-CD107A-APC antibody at 1:50 dilution (Biolegend). AD169 infected cells (MOI = 2, 96 h) were detached from plates using 10 mM EDTA and washed and resuspended 3× with complete media and then seeded onto 96 well U bottom tissue culture treated plates (Corning) at 10^5^ cells per well (viability >90%). SM5–1 Fc variants were diluted in complete medium and added to the AD169 infected wells. Finally, NK-92 cells were added to the wells and allowed to incubate at 37°C/5% CO_2_ for four hours. Controls included non-incubated NK-92 cells. Cells were spun down at 300xg and washed 3× with cold 1% FBS + PBS. Cells were resuspended in cold PBS and were analyzed using the Attune or Fortessa HTS reader. FlowJo software was used to obtain APC signals from samples and no antibody control was subtracted from each sample. Experiments were performed in independently 2–3 times with technical replicates in each set.

For assays with primary *ex vivo* NK cells, PBMC from healthy donors was isolated on histopaque and treated overnight with IFNα (1000 IU/mL). The next day, Merlin infected cells (MOI = 5, 96 h) were detached with TrypLE and seeded into a 96 well U-bottom plate at 25,000 cells/well along with 250,000 PBMC, antibodies, Golgi Stop, and CD107a-FITC (Biolegend). After 5 h, cells were stained with live/dead aqua, CD3-PeCy7, CD56-BV605, and CD57-APC (Biolegend) then fixed in 4% paraformaldehyde and analyzed on an Attune NxT flow cytometer. Experiments were performed in technical triplicate.

#### Virus dissemination assay (VDA)

SF-hTert were infected with Merlin-GFP at an MOI sufficient to achieve approximately 10 infected cells per field at 24h post-infection when imaged on an incucyte using the 10× lens. At 72 h post-infection, NK cells from the autologous donor were purified from PBMC using magnetic-activated cell sorting (human NK cell isolation kit, Miltenyi). NK cells were added to the infected cell monolayer at various effector-to-target (E:T) ratios, along with appropriate antibodies, in XVIVO15 medium.^45^ Antibodies were used at 5 μg/mL and plates incubated in an incucyte for 6–8 days and monitored every 12 h for integrated GFP intensity. As controls, each antibody was used in the absence of NK cells, and the level of control provided in the presence of NK cells normalized to this value.

#### Internalization assay followed by cell staining

Antibodies were labeled with phrodo iFL Red STP Ester (Thermo Scientific) according to manufacturer’s protocol. They were then buffer exchanged into PBS using the Superdex S200 column (GE Healthcare) on AKTA Pure FPLC (GE Healthcare) and concentrated using Amicon Ultra-30 (Millipore). AD169 infected cells (MOI = 2, 96 h) were detached from plates using 10 mM EDTA and washed and resuspended 3× with complete media (DMEM, 10% FBS) and then seeded onto 96-well U bottom tissue culture treated plates (Corning) at 10^5^ cells per well (viability >90%). Antibodies were diluted in complete media and incubated with AD169 infected cells for 2 h at 37°C and 5% CO_2_. Cells were then washed 3 times in wash buffer prior to addition of goat-*anti*-human Fcγ-AF647 (Jackson Immuno-Research, 109–606-170) secondary at a 1:500 dilution. Staining was performed on ice for one hour prior to washing 3 times and reading samples on the Fortessa using the HTS plate reader. FlowJo software was used to obtain AF647 and FITC signals from samples and stained-no antibody control was subtracted from each sample. Experiments were performed at least three times with technical replicates in each set.

#### Affinity determination using surface plasmon resonance (SPR)

SPR was performed using BIAcore X100. Two methods were used to determine kinetics for t-gp68. The first method involved immobilizing Fc2 of a CM5 chip (Cytiva) with 200 RU of human IgG1 Fc using 10 mM sodium acetate at pH 4.0. Fc1 remained as reference channel. Receptor, t-gp68-strep-FLAG, was injected at variable concentrations (250–3 nM) with 2-fold dilutions. Blank injections (0 nM) were used to subtract curves. Association time was 180 s, while dissociation was 300 s. Regeneration was performed using 10 mM glycine at pH 1.5, while 0.5 M arginine was used as an additional wash step post-regeneration. All analysis was performed using BiaEvaluation X100 software using 1:1 binding kinetics.

A second method used immobilizing Fc2 and Fc1 of CM5 chip with 4500 RU of anti-strep Fab (clone C23.21, patent # WO2015067768). Receptors (t-gp68-twin strep-FLAG and gp34-M strep-FLAG) were injected through Fc2 only at a final capture response of 40 RU. Different concentrations of hu4D5 IgG1 Fc variants were injected with an association time of 180 s, while dissociation was 300 s. Regeneration was performed using 10 mM glycine at pH 1.5, while 0.5 M arginine was used as an additional wash step post-regeneration. All analysis was performed using BiaEvaluation X100 software. For gp34-M binding, 1:1 binding kinetics analysis was performed. For t-gp68, 2:1 binding kinetics and steady state kinetics analyses were performed.

#### Affinity determination using biolayer interferometry (BLI)

Equilibrium dissociation constants (K_D_) of engineered Fc variants to FcRn and FcγRs were determined by steady-state analysis on an Octet RED96e instrument. For binding measurements of FcγRs, hu4D5 IgG1 antibody variants were immobilized onto CH1-binding (FAB2G) biosensors (Sartorius Cat. #18–5125) to a response level of 3nm. Antibody-coated sensor tips were dipped into 2-fold serial dilutions of CD16A F158 and V158 at concentrations of 5000–156.25 nM and 2000 nM–62.5 nM, respectively. Loading, baseline, association, and dissociation steps were performed at room temperature in pH 7.4 PBS with 0.02% Tween 20 and 0.1% BSA. Sensor tips were regenerated using pH 1.7 10mM glycine. For measurements of FcRn K_D_, biotinylated FcRn (AcroBiosystems Cat. #FCM-HB2W4) was immobilized onto streptavidin biosensors (Sartorius Cat. #18–5019) to a response level of 0.5 nm. FcRn-coated sensor tips were dipped into 2-fold serial dilutions of hu4D5 Fc variants from 1000 to 31.2 nM in pH 6.0 and 7.4 PBS with 0.02% Tween 20 at room temperature. Sensor tips were regenerated between cycles using pH 7.4 PBS with 0.02% Tween 20. All experiments were performed in duplicate. An unloaded tip and 0 nM analyte control were subtracted from response curves prior to analysis of steady-state kinetics by fitting one-site specific binding curves in Graphpad Prism analysis software.

#### Pharmacokinetics in huFcRn transgenic mice

All animal procedures were performed in a facility accredited by the Association for Assessment and Accreditation of Laboratory Animal Care International in accordance with protocols approved by UT Austin (AUP-2018–00093 and AUP-2022–00229). Animal Care and Use Committees and the principles outlined in the *Guide for the Care and Use of Laboratory Animals*.

Pharmacokinetic studies were performed in transgenic homozygous huFcRn Tg32 mice (Jackson Laboratory Cat #014565). Mice were administered 2 mg/kg of hu4D5 antibody Fc variants at 5–8 weeks of age by intraperitoneal injection. Blood from the lateral tail vein was collected every 3–4 days over a one-month period, except for IHH which was collected 6 times over a 48-h period. Serum concentration of antibody was determined by ELISA as follows: high-binding 96-well plates (Corning Cat# 9018) were coated with 0.5 μg/mL of chimeric Her2-Fc (R&D Systems Cat #1129-ER) antigen. Plates were blocked with 5% milk in PBS with 0.1% Tween and incubated with diluted serum samples (1:400–1:25 depending on the time point) or known concentrations of hu4D5 antibody diluted with 1:100 un-injected mouse serum in duplicate. Bound hu4D5 antibodies were detected by incubation with goat anti-human kappa light chain antibody-HRP (Southern Biotech Cat #2060–05, 1:2000 dilution). Absorbance at 450nm was measured after application of TMB substrate (Thermo Scientific Cat #34021) and neutralization with 1N HCl. Standard curves with purified hu4D5 antibody were generated for each ELISA plate and fit to a 4PL curve in Excel to quantify hu4D5 antibody present in serum samples. The β-phase elimination constant $\left(k_{e}\right)$ was determined by log-linear regression of the concentration data, including at least three time points with measurable concentrations. The beta-elimination half-lives were determined from $t_{1/2}=ln2/k_{e}$ and are shown in Table S1.

#### Negative-stain electron microscopy (nsEM)

Purified complexes of human IgG Fc bound to gp34-M and t-gp68 or gp34-M only were diluted to a concentration of 0.02–0.03 mg/mL using 2 mM Tris pH 8.0, 200 mM NaCl and 0.02% NaN_3_. Sample dilutions were performed immediately before depositing on plasma cleaned CF-400 grids (EMS) and stained using uranyl acetate (neutralized to pH 7). Grids were imaged at a nominal magnification of 60,000× (corresponding to a calibrated pixel size of 3.6 Å/pix) in a JEOL 2010F TEM microscope equipped with a Gatan OneView Camera. CTF estimation and particle picking were performed in cisTEM^78^ and extracted particles were exported to cryo-SPARC v2^79^ for 2D classification and ab initio 3D reconstruction and heterogeneous refinement.

#### Western blots

Human MRC-5 cells were infected with 5 PFU/cell (MOI = 5) of HCMV AD169 WT or the vFcγR-deletion variant Δ4. At 72 h post-infection, cells were washed once with PBS and lysed using NP40-containing buffer (140 mM NaCl, 5 mM MgCl_2_, 20 mM Tris pH 7.6 and 1% NP40). Cell debris was sedimented at 13000 rpm, 20 mins, 4°C. A sample of each cleared lysate (as indicated) was taken for subsequent western blot expression analysis. Proteins were separated by 10% SDS-PAGE and transferred to nitrocellulose membrane. Western Blot was performed with mouse anti-IE1 (1:1000; Argene, #11–003) or mouse anti-gB (SM5–1, 1μg/mL), followed by goat-*anti*-mouse Ig-HRP (Jackson Immuno Research # 115–035-003). Proteins were visualized using ECL chemiluminescence system (Li-Cor Odyssey, Germany). Membranes were re-blotted staining for β-actin as loading control (mouse anti-β-actin, Sigma #A2228).

#### Cell staining of HCMV infected cells

Human MRC-5 cells were infected with an MOI = 5 of AD169 or vFcγR-deletion variant Δ3. At 72 h post infection, cells were harvested using Accutase (Sigma-Aldrich) to retain surface molecules upon detachment from 6 well plates. Harvested cells were washed twice in 3% FBS+PBS and centrifuged at 1000xg and 4°C for 3 min. Cells were then incubated with staining buffer containing either mouse anti-gB (SM5–1; 12 μg/mL), Cytotect (1:50 dilution from a 50 mg/mL stock) or no antibody. Unconjugated primary antibody was stained using secondary antibody as indicated goat-*anti*-mouse Fcγ AF647 (1:500 of a 1 mg/mL stock; Jackson ImmunoResearch #115–605-071) or goat-*anti*-human Fcγ F(ab)_2_ AF647 (1:100 dilution of a 0.75 mg/mL stock; Jackson Labs ImmunoResearch Cat #109–606-170). Further incubation steps were carried out at 4°C for 1 h and followed by three washing steps in staining buffer. Analysis was performed on a Fortessa instrument (BD Bioscience) and data were analyzed with FlowJo (BD Bioscience).

#### Confocal microscopy

On day 0, 5×10^4^ MRC-5 cells were seeded on tissue culture treated 24-well plates with coverslip bottoms (Cellvis, #P24–1.5P). The next day, MRC-5 were infected with AD169/GFP at an MOI of 2 and allowed to incubate at 37°C/5% CO_2_ for 96 h. Antibodies were labeled with AF647 (Thermo Fisher) and buffer exchanged into PBS to remove excess dye prior to co-incubation with infected cells for 2 h at 37°C/5% CO_2_. Cells were then washed 3× with PBS and fixed with 4% paraformaldehyde for 20 min at room temperature, washed again, and then mounted on slides with DAPI-fluoromount-G (Southern Biotech). Images were collected with Zeiss LS < 710 confocal microscope (Carl Zeiss, Inc) and processed using Fiji software.

#### Static light scattering

Prior to static light scattering (SLS) measurements, samples were run on size exclusion chromatography (S200) and correct sizes were isolated and concentrated using Amicon Ultra-10 centrifugal spin columns (Millipore) to at least 10 mg/mL. SLS measurements as a function of concentration were performed at room temperature with a laser wavelength λ=658nm and a scattering angle of 90° using the miniDAWN TREOS from Wyatt Technology (Santa Barbara, CA) run in batch mode with the microcuvette accessory. The scattering intensity was allowed to stabilize at equilibrium value and then recorded and averaged over a period of ~1 min. The data was collected and processed using the Astra 6.1.2 software (Wyatt Technology, Santa Barbara, CA). The molecular weight can be determined from scattering measurements in the dilute limit using the equation:

$$\frac{K_{C}}{R_{\theta }}=\frac{1}{M_{w}}+2B_{22}C,$$

where $R_{\theta }$ is the excess Rayleigh ratio calculated from scattering intensity, $M_{w}$ is the molecular weight, $B_{22}$ is the second virial coefficient, and K is an optical constant given by:

$$K=\frac{4\pi^{2}n^{2}}{N_{A}\lambda^{4}}{\left(\frac{dn}{dc}\right)}^{2}$$

Here, $N_{A}$ is Avogadro’s number, dn/dc is the refractive index increment due to protein molecules, assumed for simplicity to remain constant at 0.185 mL/g, while $n=n_{o}+(dn/dc{)}^{*}C_{\text{prot}}$ is the solution refractive index (RI) calculated using the solvent RI ($n_{o}=1.331$ for PBS).

### QUANTIFICATION AND STATISTICAL ANALYSIS

#### Binding studies

BLI and SPR experiments were conducted with at least duplicate measurements and presented as the mean ± SEM of the indicated number of replicates. Details stated in the figure legends and detailed methods section.

#### Statistical analyses

Software used for statistical analyses described were performed with GraphPad Prism (v8.0.2). Details of the statistical models used are in each figure legend.

## Supplementary Material

1

SUPPLEMENTAL INFORMATION

Supplemental information can be found online at https://doi.org/10.1016/j.celrep.2025.116593.

## Figures and Tables

**Table 1.** Binding affinities of Fc variants for viral FcγRs measured by SPR

Ligand	Analyte	*k*_on_ (×10^5^) (M^−1^,s^−1^)	*k*_off_ (s^−1^)	Kinetic K_D_ (nM)	χ^2^	Fold change kinetic K_D_	Equilibrium K_DD_ (nM)	Fold change equilibrium K_D_

t-gp68	WT	0.40 ± 0.01	0.005 ± 0.001	110 ± 4	0.500 ± 0.05	–	–	–
WT^a^	t-gp68	0.65 ± 0.02	0.004 ± 0.010	69.0 ± 0.3	1.10 ± 0.01	1	80 ± 3	1
G5^a^	t-gp68	0.10 ± 0.01	0.021 ± 0.002	2100 ± 200	7.2 ± 0.2	30	850 ± 1	11
G2^a^	t-gp68	0.07 ± 0.01	0.032 ± 0.003	4800 ± 700	3.50 ± 0.06	70	1800 ± 1	23
WT	gp34-M	0.53 ± 0.01	0.0004 ± 0.0001	7.3 ± 0.2	1.0 ± 0.1	1	–	–
G5	gp34-M	0.37 ± 0.04	0.001 ± 0.010	33.0 ± 0.2	1.0 ± 0.1	4.5	–	–
G2	gp34-M	0.14 ± 0.01	0.004 ± 0.010	320 ± 2.0	0.4 ± 0.1	44	–	–

a For gp68 binding, 2:1 fits were used but only the first kinetic constants (k_a1_ and k_d1_) were used to determine K_D_. All antibodies expressed with hu4D5 Fab arms; WT does not include Fab arms. Dashed lines indicate unmeasured data. Fold-change values determined as K_D_(variant)/K_D_(WT).

**Table 2.** Binding affinities of Fc variants for host FcγRs measured by BLI

	Equilibrium K_D_, nM			
	
	FcRn pH 6.0	FcRn pH 7.4	CD16A V158	CD16A F158

WT	50 ± 20	ND	170 ± 10	1160 ± 10
G5	90 ± 10	ND	150 ± 10	990 ± 10
G2	40 ± 10	ND	120 ± 20	360 ± 10

ND: not-determined kinetics due to low signal.

**KEY RESOURCES TABLE:** 

REAGENT or RESOURCE	SOURCE	IDENTIFIER

Antibodies		

Cytogam	Dell Medical School Pharmacy	N/A
Cytotect	Apotheke Hospital	N/A
Goat anti human κ HRP	Southern Biotech	Cat# 2060-05
Goat anti human Fcγ AF647	Jackson Immuno-research	Cat# 109-606-170
Goat anti mouse Fcγ AF647	Jackson Immuno-research	Cat# 115-605-071
Goat anti mouse IgG HRP	Southern Biotech	Cat# 1010-05
Goat anti-human IgG-POD	Jackson ImmunoResearch	Cat# 109-035-003
Goat anti-mouse POD	Jackson ImmunoResearch	Cat# 115-035-003
Goat anti-rabbit IgG	Sigma Aldrich	Cat# A6154-1ML
Human Fab_2_ anti-strep-tag (clone C23.21)	patent # WO2015067768	N/A
Mouse anti β-actin,	Sigma-Aldrich	Cat# A2228
Mouse anti FLAG (M2) HRP	Sigma-Aldrich	Cat# A8592
Mouse anti FLAG (M2) PE	Biolegend	Cat# 637309
Mouse anti human HLA-A,-B,-C, APC	Biolegend	Cat# 311400
Mouse anti human HLA-A2, APC	Biolegend	Cat# 343308
Mouse anti IE1	Argene	Cat# 11-003
Mouse anti LAMP1 (CD107A) APC	Biolegend	Cat# 328620
Panel of human IgG1 Fab and Fc variants	This study	N/A
Panel of mouse IgG2A Fab variants	This study	N/A
Rabbit anti-human IgA	Jackson ImmunoResearch	Cat# 309-005-011

Virus strains		

HCMV AD169/GFP	Yu et al.^71^	N/A
HCMV AD169varL	Le et al.^72^	GenBank: MN900952.1
HCMV AD169varL-Δ3	Kolb et al.^23^	N/A
HCMV Merlin	Stanton et al.^73^	GenBank:GU179001.
HCMV Merlin	Stanton et al.^73^	GenBank:GU179001.
HCMV Merlin Δ4	Vlahava et al.^32^	N/A
HCMV Merlin Δ4	Vlahava et al.^32^	N/A
HCMV Merlin-GFP	Nightingale et al.^74^	N/A
HCMV Merlin-GFP	Nightingale et al.^74^	N/A
HCMV TB40/E	Sinzger et al.^75^	N/A

Chemicals, peptides, and recombinant proteins		

AD169 gp34 wild-type or C139S (aa# 24–182)	This study	N/A
AD169 t-gp68 (aa# 68–289)	This study	N/A
Alexa Fluor 647 Protein Labeling Kit	Fisher Scientific	Cat# A20173
Avidin	Sigma Aldrich	Cat# A9275-25MG
BD GolgiPlug	BD Pharmingen	Cat# 555029
BD GolgiStop	BD Pharmingen	Cat# 554724; RRID: AB_2869012
Biotin	Sigma Aldrich	Cat# B4501-10G
Biotinylated human CD16A V158	Sino Biological	Cat# 10389-H27H1B
Biotinylated human FcRn	ACROBiosystems	Cat# FCM-H82W4
Calcein AM	BD Pharmingen	Cat# 564061
CD16A F158 ectodomain, His tag	SinoBiological	Cat# 10389-H08H
CD16A V158 ectodomain, His tag	SinoBiological	Cat# 10389-H27H
Flash Red 1μm Beads	Bangs Laboratories	Cat# FSFR004
HCMV gB post-fusion	Ye et al.^76^	N/A
HCMV gB, recombinant from Towne	SinoBiological	Cat# 10202-VCCH1
Her2-Fc	R&D Systems	Cat# 1129-ER
Human CD16A-F158	This study	N/A
Human CD16A-GST	This study	N/A
Human CD16A-V158	This study	N/A
Human FcRn-GST	This study	N/A
pHrodo iFL Green STP Ester	Thermo Fisher Scientific	Cat# P36013
pHrodo iFL Red STP Ester	Thermo Fisher Scientific	Cat# P36011
Streptavidin AF647	Jackson ImmunoResearch	Cat# 016600084
Streptavidin PE	Biolegend	Cat# 405204

Critical commercial assays		

ExpiFectamine 293 Transfection Kit	Thermo Fisher Scientific	Cat# A14524
ExpiFectamine CHO Transfection Kit	Thermo Fisher Scientific	Cat# A29129
Lipofectamine 2000	Thermo Fisher Scientific	Cat# 11668019

Experimental models: Cell lines		

BeWo b30 syncytiotrophoblast cell line	ATCC	Cat# CLL-98
BW5147 mouse thymoma cells	Hadassah Hospital	N/A
CHO-K1	ATCC	Cat# CCL-61
*Ex vivo* NK cells	Local Donors	N/A
Expi293	Thermo Fisher Scientific	Cat# A41249
ExpiCHO	Thermo Fisher Scientific	Cat# A29133
HFF fibroblast cells	ATCC	Cat# SCRC-1041
HFFF-hTert	Stanton et al.^73^	N/A
MRC5	ATCC	Cat# CCL-171
NK-92 F158	ATCC	Cat# PTA-8837
NK-92 V158	ATCC	Cat# PTA-8836
Skin Fibroblasts	Local Donors	N/A
SKOV3	ATCC	Cat# HTB-77
THP-1	ATCC	Cat# TIB-202

Experimental models: Organisms/strains		

DH5α electrocompetent cells	NEB	Cat# C2987H
EBY100	ATCC	Cat# MYA-4941
Human FcRn transgenic mice (FcRn−/− hFcRn (32) Tg mice	The Jackson Laboratory	Cat# 014565

Oligonucleotides		

Primers for Fc cloning into yeast	Table S2	N/A
Primers for gp34 and gp68 cloning	Table S2	N/A
Primers for IgG1 cloning	Table S2	N/A
Primers for generating AD169 mutants	Table S2; Kolb et al.^23^	N/A

Recombinant DNA		

AbVec1.1-27287-IGKC	This study	N/A
AbVec1.1-2B1-IGKC	This study	N/A
AbVec1.1-hu4D5-IGKC	This study	Bostrom et al.^77^
AbVec1.1-SM51-IGKC	This study	N/A
AbVec2.0-27287-IGHG1	This study	N/A
AbVec2.0-2B1-IGHG1	This study	N/A
AbVec2.0-2B1-IGMG2A	This study	N/A
AbVec2.0-hu4D5-IGHG1	This study	N/A
AbVec2.0-SM51-IGHG1	This study	^3030^
pcDNA3.0 ΔNT gpRL13 FLAG-PDGFRα	This study	N/A
pcDNA3.0 gp34 FLAG-PDGFRα	This study	N/A
pcDNA3.0 gp34 FLAG-strep	This study	N/A
pcDNA3.0 gp34-C139S FLAG-strep	This study	N/A
pcDNA3.0 gp68 FLAG-PDGFRα	This study	N/A
pcDNA3.0 gpRL12 FLAG-PDGFRα	This study	N/A
pcDNA3.0 human IgG1 Fc	This study	N/A
pCDNA3.0 SM5-1 IGMG2A	This study	N/A
pcDNA3.0 t-gp68 FLAG-strep	This study	N/A
pCTcon2	Addgene	RRID: Addgene_41843
pCTcon2 human CH_2_-CH_3_	This study	N/A

Software and algorithms		

Astra Software V6.1.2	Wyatt Technology	RRID:SCR_016255
Biacore X100 Evaluation Software	GE Healthcare	V2.0.1
cisTEM	Grant et al.^78^	RRID: SCR_016502
cryoSPARC	Punjani et al.^79^	RRID: SCR_016501
Fiji	Schindelin et al.^80^	RRID: SCR_002285
FlowJo	BD Bioscience	RRID: SCR_008520
GraphPad Prism	Motulsky et al.^81^	RRID: SCR_002285
Image studio Luie, v5.2	LICORbio	N/A
Octet Data Analysis Software	Forte Bio	V11.1
ViiA 7 Software	Thermo Fisher Scientific	N/A

Other		

HBS EP + buffer	Cytiva	Cat# BR100669
HiTrap Protein A columns	Cytiva	Cat# 17-5498-54P
IMAC Sepharose 6 Fast Flow resin	Cytiva	Cat# 17092107
Octet Anti-Human Fab-CH1 2ND Generation (FAB2G) Biosensors	Forte Bio	Cat# 18-5125
Octet Streptavidin (SA) Biosensor	Forte Bio	Cat# 18-5019
Protein Thermal Shift Dye Kit	Thermo Fisher Scientific	Cat# 4461146
Series S Sensor Chip CM5	Cytiva	Cat# BR100530
Strep-Tactin XT Superflow high-capacity cartridge	IBA	Cat# 2-4026-001
Superdex 200 Increase 10/300 GL	Cytiva	Cat# 28-9909-44
TMB Substrate	Thermo Fisher Scientific	Cat# 34021

## Highlights

- Host and HCMV FcR compete for IgG1 binding but engage different residues
- Fc-engineering abrogates viral FcR antagonism while retaining CD16A activation
- Antibodies that resist vFcγR capture promote superior ADCC against infected cells
- Designer Fc domains complement Fabs to create enhanced disease-specific therapies
