# Supplementary material for: Selective decoupling of IgG1 binding to viral Fc receptors restores antibody-mediated NK cell activation against HCMV
Source: Cell Rep. Author manuscript; Available in PMC 2026 Jan 24. (PMC12831531; doi:10.1016/j.celrep.2025.116593)
Supplement: 1 [file NIHMS2132727-supplement-1.pdf]

Cell Reports, Volume *44*

Supplemental information

Selective decoupling of IgG1 binding

to viral Fc receptors restores antibody-mediated

NK cell activation against HCMV

Ahlam N. Qerqez, Katja Hoffmann, Alison G. Lee, Sumit Pareek, Kelli Hager, Akaash K. Mishra, George Delidakis, Kirsten Bentley, Lauren Kerr-Jones, Mica Cabrera, Truong Nguyen, Rebecca L. Göttler, Amjad Chowdhury, Philipp L. Kolb, Hartmut Hengel, George Georgiou, Jason S. McLellan, Richard J. Stanton, Annalee W. Nguyen, and Jennifer A. Maynard

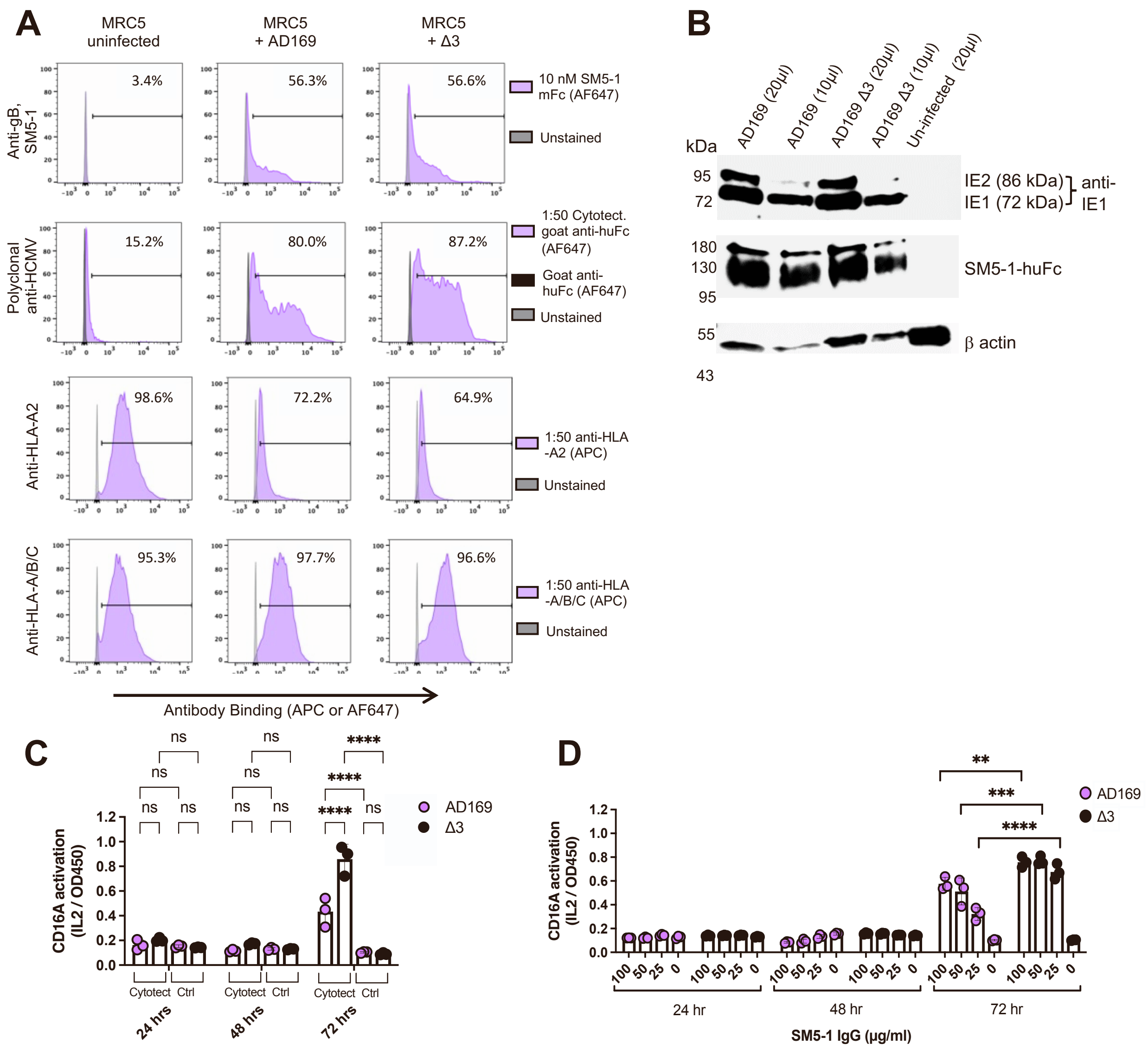

Figure S1, related to Figure 1. AD169 and Δ3 strains have similar expression of gB and other proteins. Human MRC5 fibroblasts were infected with AD169 or Δ3 virus (MOI = 5, 72 hpi), with mock-infected MRC5 cells used as controls. **A,** Cells were stained with 10 nM SM5-1-mouse Fc-AF647, 1:50 anti-HLA-A2 (APC), 1:50 anti-HLA-A/B/C (APC), or 1:50 Cytotect followed by goat-anti-human Fc-AF647. Samples were analyzed by flow cytometry, with normalized histograms shown and positive cells gated to indicate the percent positive cells. **B,** As a complementary approach to measure gB expression, Western blot was performed on cell lysate from infected cells using SM5-1-mFc to detect gB, with IE1/IE2 expression as controls for cell infection and β actin as a loading control. CD16A activation after incubation of BW-CD16A-ζ reporter cells with HCMV-infected cells (24, 48, and 72 hpi) and **C**, Cytotect or **D,** anti-gB antibody SM5-1, each at 100 μg/mL. CD16A activation was monitored by mouse IL2 secretion measured by ELISA, with data presented as mean ± SD (n=2) and repeated twice. Statistical analyses were performed by GraphPad using two-way ANOVA followed by Tukey's multiple comparison test, with *p<0.05, **p<0.01, ***p<0.001, ****p<0.0001, and ns: non-significant.

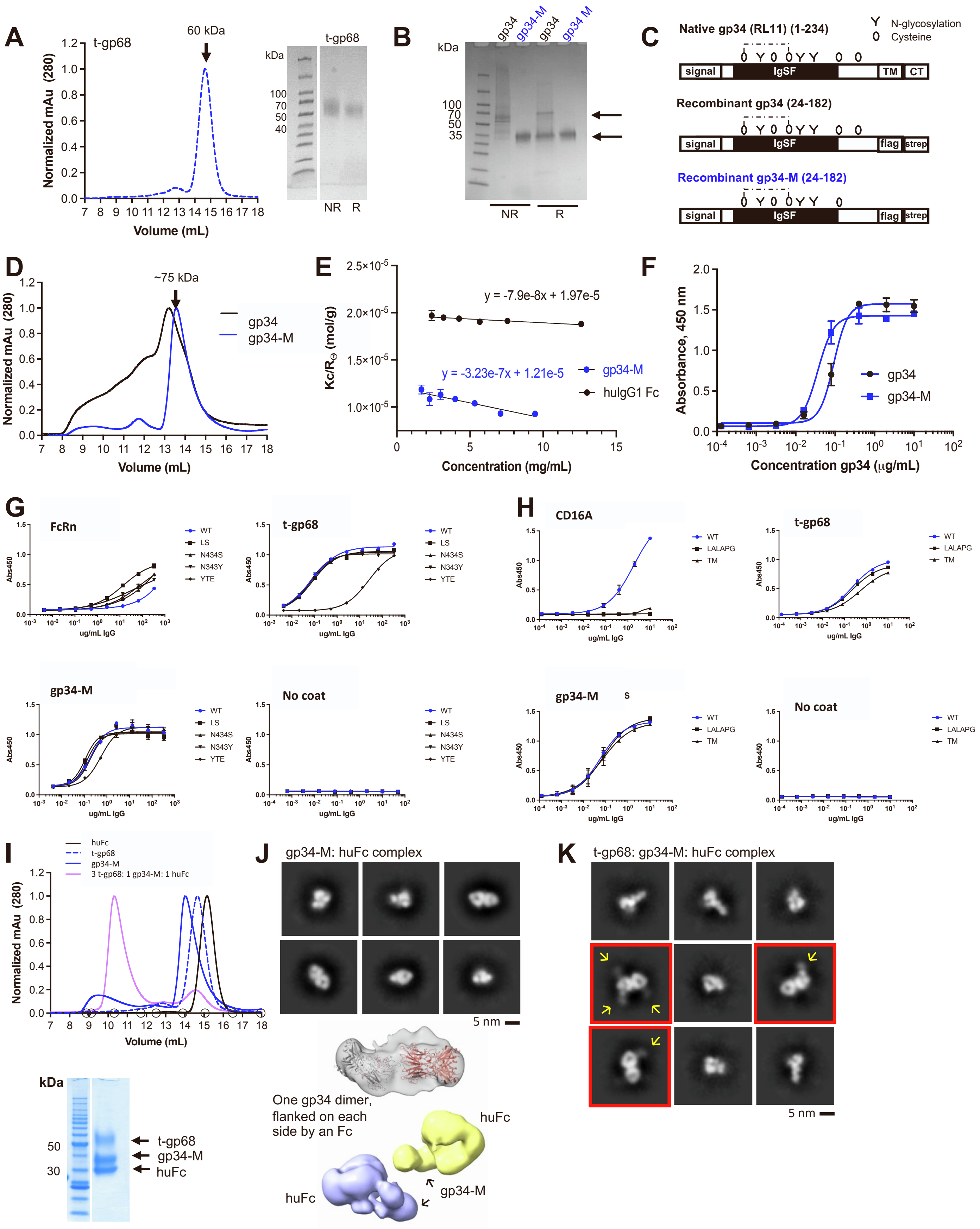

A
t-gp68
60 kDa
Normalized mAu (280)
Volume (mL)
kDa
NR R
B
gp34
gp34-M
NR
R
C
Native gp34 (RL11) (1-234)
N-glycosylation
Cysteine
signal
IgSF
TM
CT
Recombinant gp34 (24-182)
flag
strep
Recombinant gp34-M (24-182)
D
~75 kDa
gp34
gp34-M
E
y = -7.9e-8x + 1.97e-5
y = -3.23e-7x + 1.21e-5
gp34-M
huIgG1 Fc
Kc/RΘ (mol/g)
Concentration (mg/mL)
F
Absorbance, 450 nm
Concentration gp34 (μg/mL)
G
FcRn
t-gp68
gp34-M
No coat
WT
LS
N434S
N343Y
YTE
Abs450
ug/mL IgG
H
CD16A
WT
LALAPG
TM
I
huFc
t-gp68
gp34-M
3 t-gp68: 1 gp34-M: 1 huFc
J
gp34-M: huFc complex
5 nm
One gp34 dimer, flanked on each side by an Fc
huFc
gp34-M
K
t-gp68: gp34-M: huFc complex

<u>Figure S2, related to Figure 2.</u> Biochemical characterization of engineered gp68 and gp34 variants.

After strep tag purification and polishing with an S200 size exclusion column, t-gp68 and gp34 ectodomain proteins were analyzed by analytical SEC (100 µg) and 4-20% SDS-PAGE under reducing (R) and non-reducing (NR) conditions for **A,** gp68 and **B,** SDS-PAGE for gp34 and gp34-M (3 µg per lane).

C, Modifications of recombinant, truncated gp34 to remove N-linked glycosylation sites (Y) and cysteines (0) and generate gp34-M are shown, with the inferred di-sulfide bonding pattern (dashed line).

D, Monodispersity of gp34 and gp34-M was assessed by injecting purified protein (100 µg) on an S200 size exclusion chromatography column.

E, Rhe molecular weight of purified Fc and gp34-M was estimated by static light scattering (SLS) using the inverse y-intercept of the equation $Kc/R_\theta = 1/M_W + 2B_{22}c$. Fc was measured 51±0.3 kDa while gp34-M measured as 84±1 kDa.

F, Fc binding activity was assessed by ELISA with immobilized human Fc, serially diluted gp34 or gp34-M, followed by anti-FLAG (M2)-HRP detection. Representative data of one experiment is shown with each experiment repeated at least twice with technical replicates.

G, To determine whether Fc changes known to impact FcRn binding also impact vFcγR binding, ELISA plates were coated with purified host Fc receptor, t-gp68, gp34-M or no coat. After blocking, serially diluted hu4D5 antibodies with WT, LS (M428L, N434S), N434S, N434Y Fcs or YTE (M252Y, S254T, T256E) were applied (333 to 0.004 µg/mL in 5-fold dilution steps) and detected with goat-anti-kappa-HRP and 4PL fits determined using GraphPad. Representative data shown (n=2); all experiments were performed at least twice.

H, To determine whether Fc changes known to impact CD16A binding also impact vFcγR binding, similar ELISAs were performed using hu4D5 antibodies with WT, LALAPG or TM Fc domains at 10-0.0001 µg/mL in 5-fold dilution steps with detected and analysis as in G.

I, For low resolution negative-stain electron microscopy imaging of the Fc interactions with vFcγR, analytical size exclusion chromatography of t-gp68, gp34-M, Fc, and the ternary complex (3:1:1 molar ratio) was performed using an S200 column and by SDS-PAGE, with molecular weight standards indicated by circles (blue dextran, thyroglobulin, ferritin, beta amylase, aldolase, conalbumin, ovalbumin, carbonic anhydrase, cytochrome C).

J, Negative-stain EM particle analysis of SEC-purified Fc:gp34-M complexes. *Top,* particles were used to generate negative stain 2D classes showing two Fc molecules bound together by a gp34-M dimer at the CH_2 tip. *Bottom,* the 3D reconstruction from the particles indicates the extra density between the two Fc molecules corresponds to a gp34-M dimer (black arrows). Scale bar is 5 nm.

K, Negative stain EM particle analysis of SEC-purified Fc+gp34-M+t-gp68 complexes. Particles were used to generate negative stain 2D classes showing two Fc molecules bound together by a gp34-M dimer and t-gp68 appendages with partial occupancy (boxes outlined in red) protruding from the CH_2-CH_3 interface. Yellow arrows indicate partially occupied t-gp68 sites, with up to three t-gp68 molecules observed per complex. Scale bar is 5 nm.

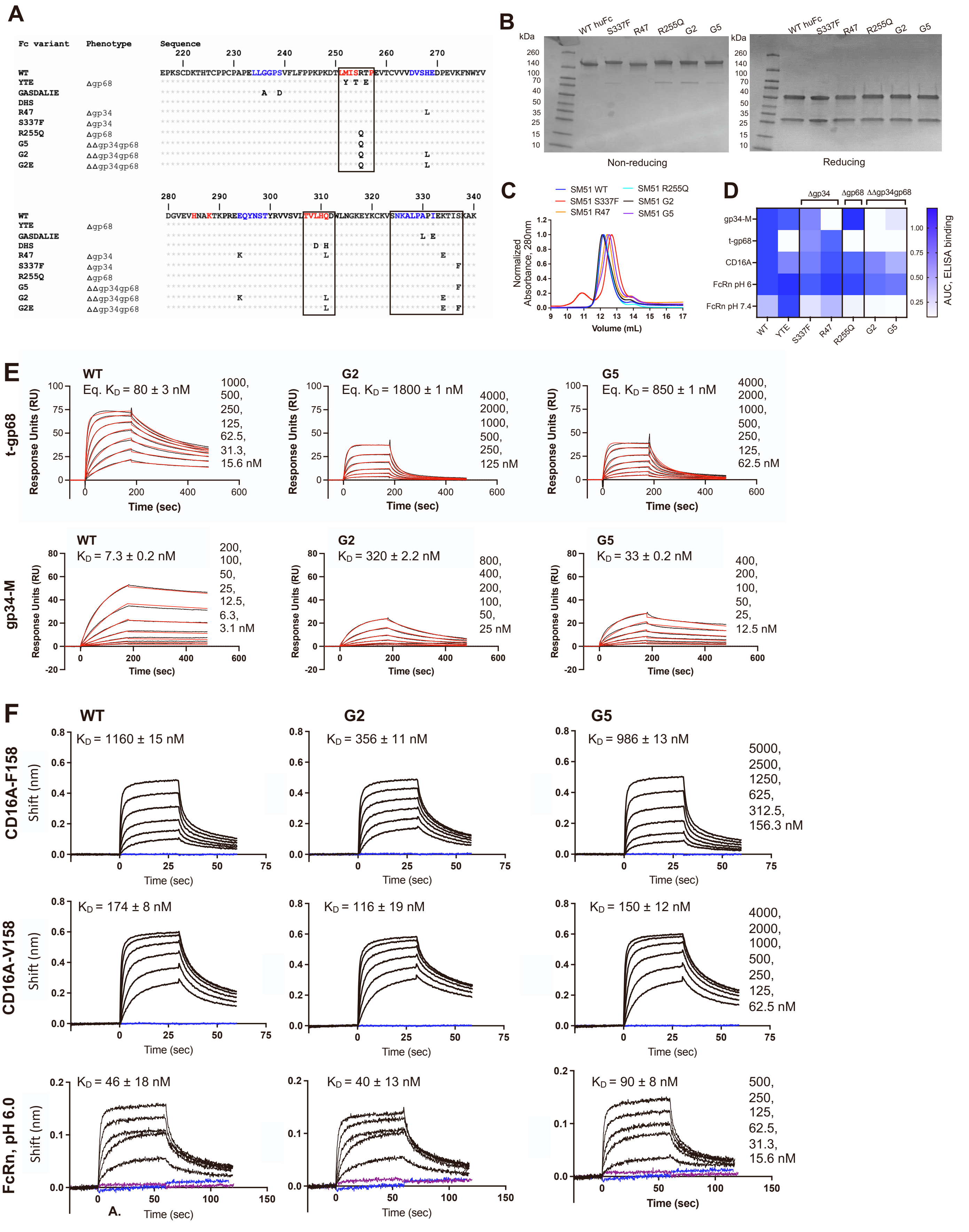

A
Fc variant
Phenotype
Sequence
220 230 240 250 260 270
EPKSCDKTHTCPPCPAPELLGGPSVFLFPPKPKDTLMISRTPEVTCVVVDVSHEDPEVKFNWYV
WT
YTE Δgp68 Y T E
GASDALIE A D
DHS
R47 Δgp34 L
S337F Δgp34
R255Q Δgp68 Q
G5 ΔΔgp34gp68 Q
G2 ΔΔgp34gp68 Q L
G2E ΔΔgp34gp68 Q L
280 290 300 310 320 330 340
DGVEVHNAKTKPREEQYNSTYRVVSVLTVLHQDWLNGKEYKCKVSNKALPAPIEKTISKAK
WT
YTE Δgp68
GASDALIE L E
DHS D H
R47 Δgp34 K L E
S337F Δgp34 F
R255Q Δgp68
G5 ΔΔgp34gp68 F
G2 ΔΔgp34gp68 K L E
G2E ΔΔgp34gp68 L E F
B
kDa
WT huFc S337F R47 R255Q G2 G5
260 140 100 70 40 50 35 25 15 10
Non-reducing
Reducing
C
SM51 WT
SM51 S337F
SM51 R47
SM51 R255Q
SM51 G2
SM51 G5
Normalized Absorbance, 280nm
Volume (mL)
D
Δgp34 Δgp68 ΔΔgp34gp68
gp34-M
t-gp68
CD16A
FcRn pH 6
FcRn pH 7.4
WT YTE S337F R47 R255Q G2 G5
AUC, ELISA binding
1.00 0.75 0.50 0.25
E
t-gp68
WT
Eq. K_D = 80 ± 3 nM
1000, 500, 250, 125, 62.5, 31.3, 15.6 nM
G2
Eq. K_D = 1800 ± 1 nM
4000, 2000, 1000, 500, 250, 125 nM
G5
Eq. K_D = 850 ± 1 nM
4000, 2000, 1000, 500, 250, 125, 62.5 nM
Response Units (RU)
Time (sec)
gp34-M
WT
K_D = 7.3 ± 0.2 nM
200, 100, 50, 25, 12.5, 6.3, 3.1 nM
G2
K_D = 320 ± 2.2 nM
800, 400, 200, 100, 50, 25 nM
G5
K_D = 33 ± 0.2 nM
400, 200, 100, 50, 25, 12.5 nM
F
WT
G2
G5
CD16A-F158
K_D = 1160 ± 15 nM
K_D = 356 ± 11 nM
K_D = 986 ± 13 nM
5000, 2500, 1250, 625, 312.5, 156.3 nM
CD16A-V158
K_D = 174 ± 8 nM
K_D = 116 ± 19 nM
K_D = 150 ± 12 nM
4000, 2000, 1000, 500, 250, 125, 62.5 nM
FcRn, pH 6.0
K_D = 46 ± 18 nM
K_D = 40 ± 13 nM
K_D = 90 ± 8 nM
500, 250, 125, 62.5, 31.3, 15.6 nM
Shift (nm)
Time (sec)
A.

Figure S3, related to Figure 3. Binding of Fc variants to gp34, gp68 and host receptors.

A, Amino acid sequence allignment of CH_2 domains for selected Fc variants with reduced binding to gp34 and gp68. Many residue changes fall within the binding epitopes of FcRn (red residues) and CD16A (blue residues). After generation of SM5-1 antibodies with Fc variants, purified proteins were analyzed by

B, 4-20% SDS-PAGE under reducing (R) and non-reducing (NR) conditions and

C, analytical SEC to assess monodispersity after injection of 100 µg onto an S200 SEC column with normalized chromatograms shown.

D, Antibodies with hu4D5 Fabs and Fc variants were used in ELISA to assess binding to FcRn-GST, CD16A-GST, gp34-M and t-gp68. Antibodies were coated at 4 µg/ml, followed by serially diluted receptors at concentrations spanning the full dose-response curve and detected with anti-FLAG-M2-HRP. The AUC was calculated using GraphPad, normalized to maximum AUC from the data set, and plotted as a heat map such that more color means more binding. This experiment was performed twice, with representative data shown.

E, To measure binding of hu4D5 Fc variants to vFcγR**,** SPR was performed with CM5 chips coupled with anti-strep Fab at 4500 RU to capture strep-tagged t-gp68 or gp34-M to a final RU of 35-40. Antibodies at the indicated concentrations were allowed to associate for 180 seconds and then dissociate for 300 seconds. Kinetics for t-gp68 was determined by 2:1 binding fits with equilibrium K_D values and gp34-M binding determined with 1:1 kinetic fits. All data were analyzed using the BIAcore X100 Evaluation software; data shown are representative of those from two repeats.

F, To measure binding of hu4D5 Fc variants to host Fc receptors, BLI was performed. To measure binding to CD16A alleles F158 and V158, CH1 binding sensors were loaded with hu4D5 Fc variants and then dipped into wells containing receptors at the indicated concentrations. For FcRn, streptavidin sensors captured the receptor, before dipping into wells with the indicated antibody concentration at pH 6.0 and the highest antibody concentration (500 nM) used to assess binding at pH 7.4 (purple). Sensograms shown are representative of two independent runs with traces lacking antibody in blue and equilibrium fits in red. Steady-state binding kinetics determined by Langmuir fit with Octet software.

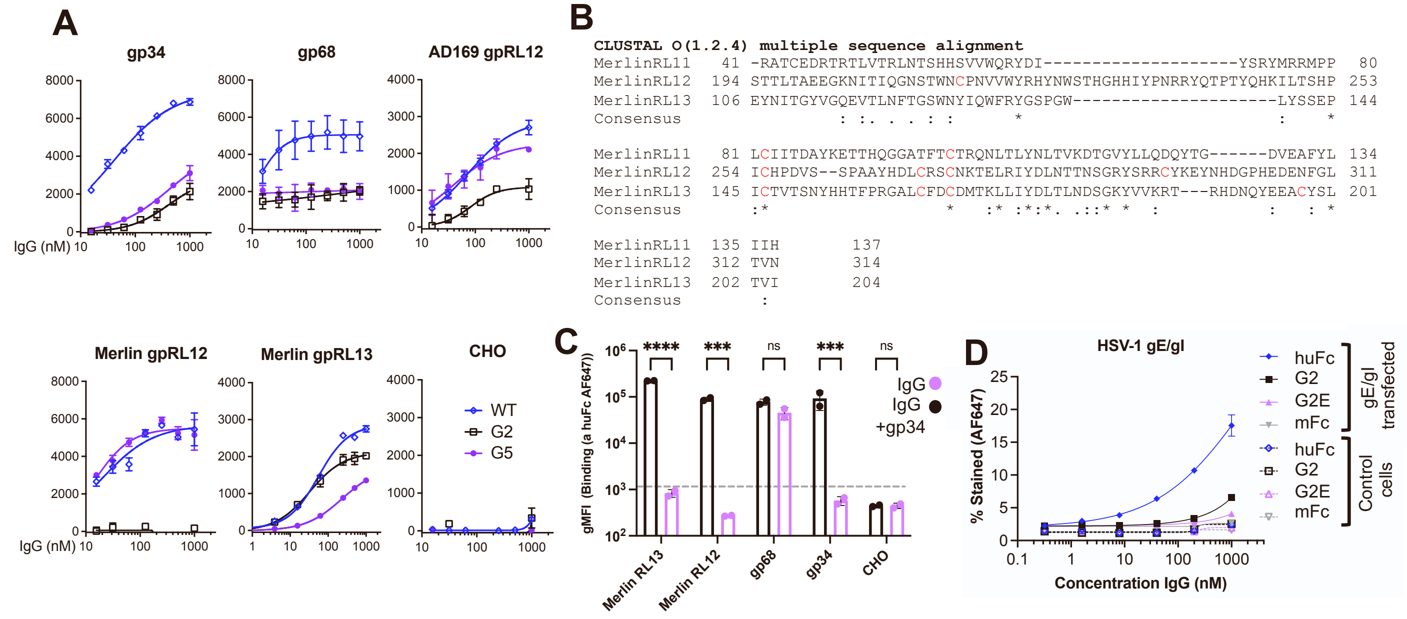

Figure S4, related to Figure 3. Binding of Fc variants to other viral Fc receptors.

A, To assess Fc binding to different vFcγRs, the extracellular domains of gp34, gp68, AD169 RL12, Merlin RL12, and Merlin RL13, were expressed on the ExpiCHO cell surface with c-terminal FLAG tags and a PDGFRα transmembrane domain and incubated with serially diluted hu4D5-Fc variants. Anti-FLAG PE staining was used to gate for vFcγR-positive cells, and goat-anti-huFcγ-AF647 used to measure the GMFI of double positive cells using flow cytometry. Non-transfected ExpiCHO cells served as negative controls. GMFI was plotted with 4PL curve fits performed in GraphPad.

B, Clustal Omega protein sequence alignment of the predicted Ig-fold domains of Merlin *RL11, 12,* and *13* show less than 20% homology; Ig-fold sequences were predicted using the SMART data bank.

C, To assess the ability of gp34 to inhibit Fc binding to different vFcγRs, ExpiCHO cells expressing Merlin RL12, RL13, gp34, or gp68 were stained with 300 nM hu4D5 with WT Fc alone or in the presence of 3000 nM soluble gp34-M before measuring bound antibody by flow cytometry, as in G.

D, To assess whether the Fcs identified here also confer resistance to capture by vFcγRs expressed by HSV-1, these gE and gI ectodomains were expressed on ExpiCHOs and incubated with serially diluted antibodies comprised of isotype control 2B1 and Fc variants. Bound antibodies were detected by goat-F(ab)'2 anti-FcKappa-AF647 and 4PL curves fitting with GraphPad, with non-transfected cells used as specificity controls. For G, I and J, representative data are shown, with each experiment was repeated at least twice. One-way ANOVA with Tukey test multiple comparisons used to assess significance. *$p<0.05$, **$p<0.01$, ***$p<0.001$, ****$p<0.0001$, and ns: non-significant.

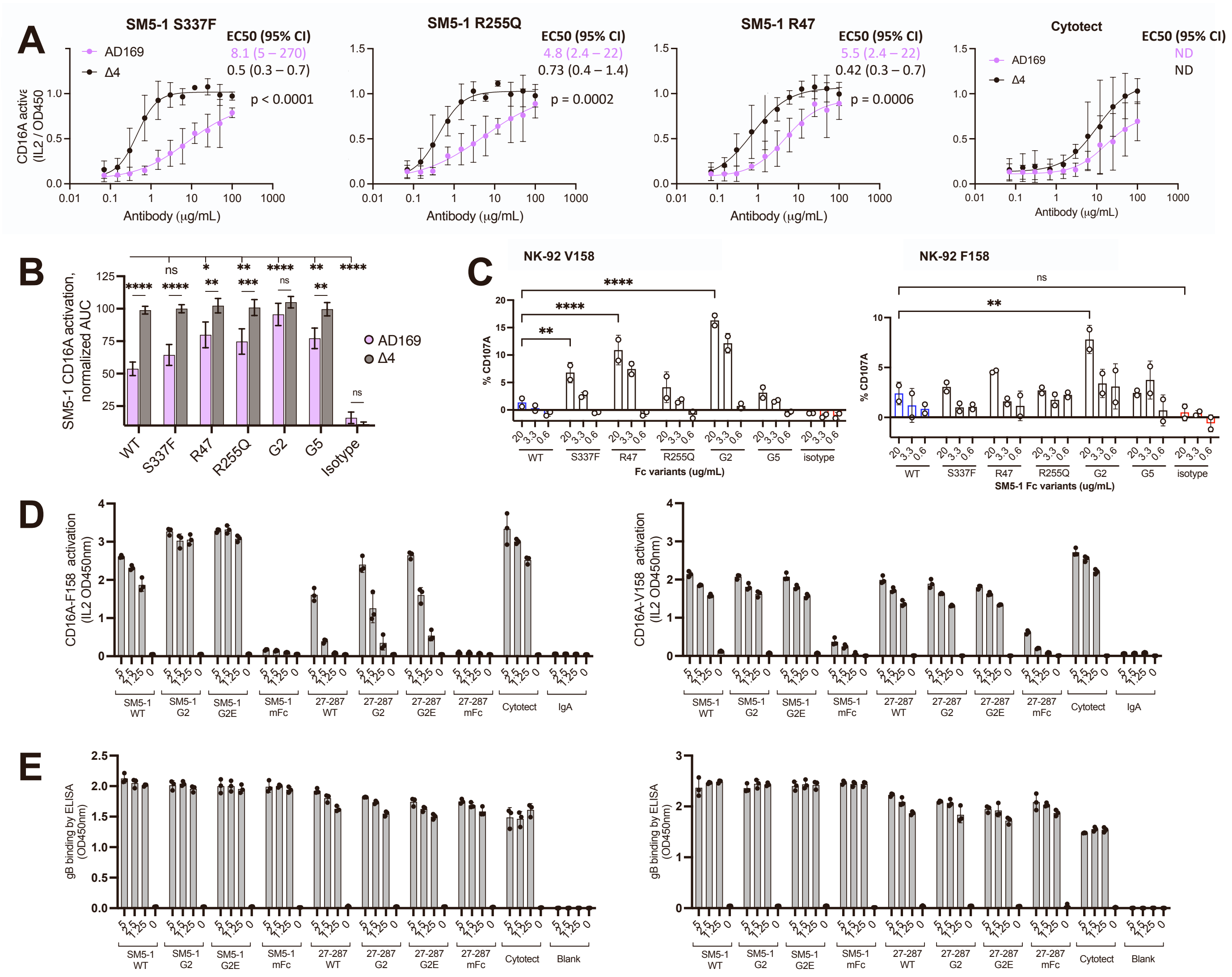

<u>Figure S5, related to Figure 4.</u> Biological characterization of SM5-1 Fc variants.

A. To compare CD16A activation for different Fc variants, BW-CD16A-ζ reporter cells were incubated with Fc variants SM5-1 antibodies (100-0.07 μg/mL; two-fold dilutions) and AD169- or Δ3-infected fibroblasts (MOI=5, 72 hpi), and CD16A activation measured by mIL-2 secretion. Data were fit to 4PL curves, with the mean EC_{50} values (μg/mL) for each antibody noted; ND indicates activation was not detected at 100 μg/mL. The written p-values indicate EC_{50} differences for one antibody with AD169- versus Δ3-infected cells; asterisks indicate the p-value for variant versus WT EC_{50} (shown in Fig. 4).

B, AUC for each antibody normalized to the AUC for WT with Δ3 infection. Data shown are the mean±SD of three independent experiments.

C, The percent of CD107+ degranulated NK-92 cells after incubation with SM5-1 antibodies (20, 3.3, 0.6 μg/mL) and AD169-infected fibroblasts (MOI=2, 96 hpi) measured by flow cytometry. Mean ± SD (n=2) shown with significance determined by two-way ANOVA with Tukey's multiple comparisons test. To assess whether Fc variants enhance

D, CD16A activation or **E,** gB binding, immobilized gB was incubated with SM5-1 antibodies (5 μg/ml with 2-fold dilutions) with BW-CD16A-ζ reporter cells expressing human CD16A-V158 or CD16A-F158 added and CD16A activation measured by secreted and anti-gB by adding goat-anti human IgG-HRP or goat anti-mouse IgG-HRP. Data are mean of n=3 replicates from one representative experiment.

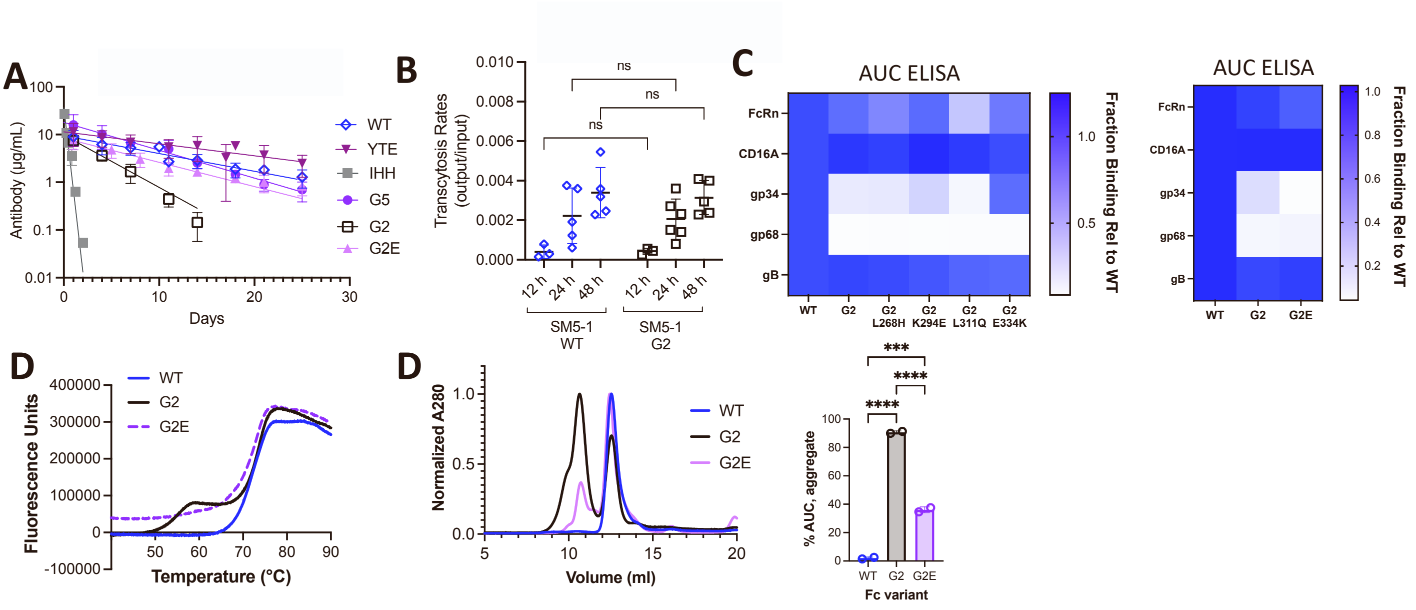

<u>Figure S6, related to Figure 4.</u> Development of Fc variant G2E using SM5-1 Fc variants.

A, To assess in vivo pharmacokinetics, Tg32 mice expressing human FcRn were administered 2 mg/kg of one hu4D5 antibody intra-peritoneally. Sera concentrations were measured at indicated time points with the mean ± SD shown (n=3-7). Data for each mouse were fit to a single exponential decay model in Graphpad, with the half-life calculated as $t_{1/2}$ = ln2/ beta-elimination rate and averaged for mice receiving the same antibody and shown in Table S2.

B, Transcytosis kinetics measured using the BeWo b30 syncytiotrophoblast cell line, with antibody levels quantified by ELISA, with data presented as mean ± SD (n=5). One-way ANOVA with Tukey's multiple comparisons was used for statistical analysis.

C, ELISA evaluated the receptor binding profile of SM5-1 G2 reversion variants using immobilized antibody, with serially diluted receptors (pH 7.4, pH 6.0 for FcRn) added and detected with goat-anti-FLAG-HRP. Data were fit to 4PL curves and AUC determined and normalized to wild-type Fc for each receptor to plot as a heat map. Representative data shown (n=2), all experiments performed at least twice.

D, Stability of SM5-1 Fc variant antibodies assessed by differential scanning fluorimetry to measure melting temperature (Tm) and

E, analytical SEC (S200 column) to monitor higher-molecular weight aggregates after incubation of 1 mg/ml antibody at 50°C for 24 hrs. The AUC of the larger left peak was quantified and plotted to compare aggregation. One-way ANOVA with Tukey's multiple comparisons was used for statistical analysis. All analyses performed in GraphPad with *p<0.05, **p<0.01, ***p<0.001, ****p<0.0001, ns = non-significant.

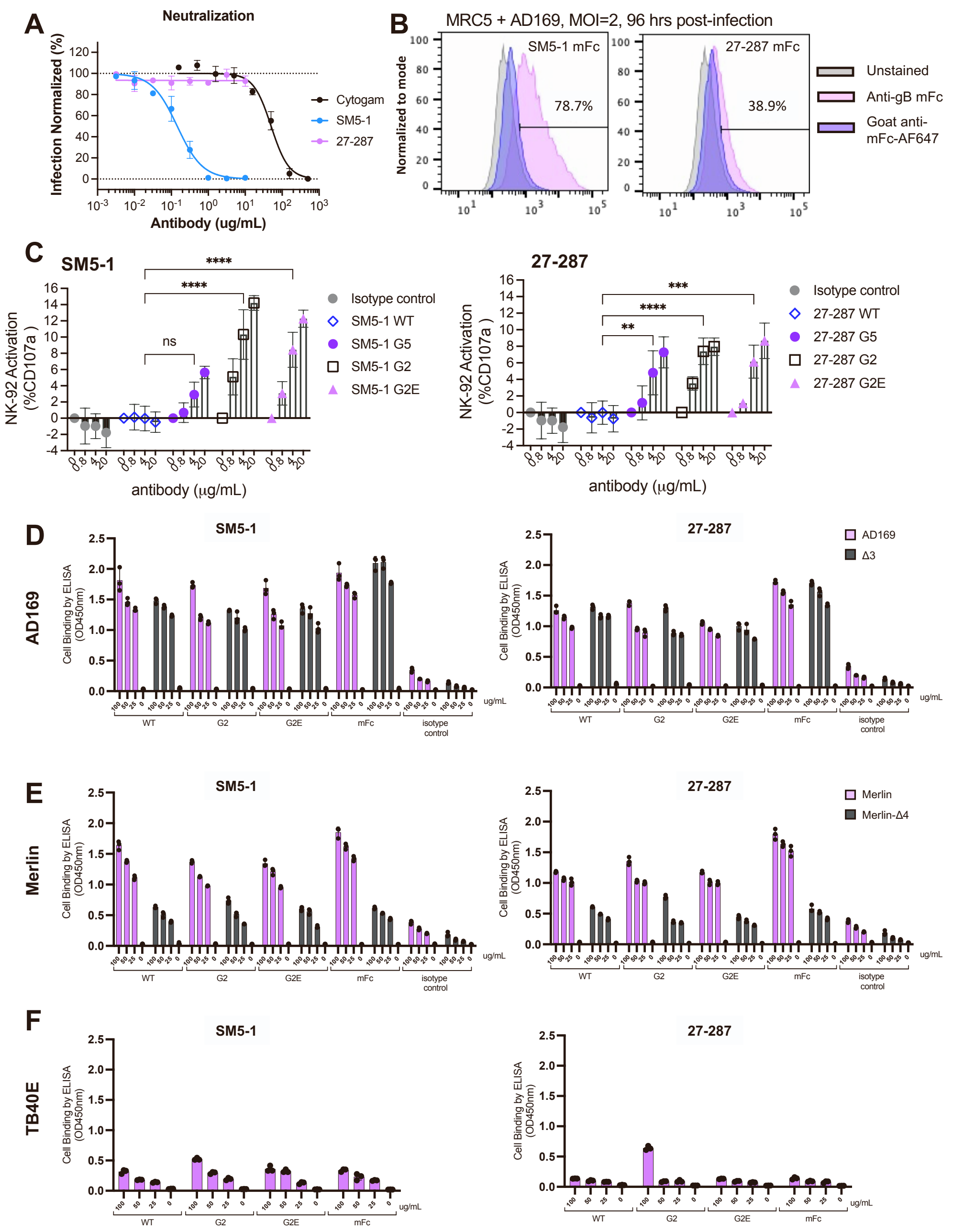

<u>Figure S7, associated with Figure 5.</u> Antibody 27-287 and SM5-1 and HCMV infection characterization. A, To compare neutralization ability in the absence of complement, AD169 virions were incubated with Cytogam, SM5-1 and 27-287, all with WT Fc before combining with MRC-5 cells and measuring viral infectivity 24 hours later. Data were normalized to no antibody (100%) and uninfected (0%) controls within each plate and are presented as mean ± SD (n=2) for n=2 experiments. **B,** To compare epitope accessibility, AD169-MRC5 infected cells (MOI= 2, 96 hpi) were stained with 27-287-mFc or SM5-1-mFc antibodies, with antibody binding measured by flow cytometry. **C,** To compare antibody ADCC, the percent of human NK-92 cells (CD16A V158) exhibiting CD107A-positive degranulation after incubation with AD169-infected MRC5 (MOI=2, 96 hpi) and SM5-1 or 27-287 antibodies with different Fcs are shown. Data presented as mean ± SD (n=2) and average of n=3 experiments, with two-way ANOVA with Tukey's multiple comparisons test and *p<0.05, **p<0.01, ***p<0.001, ****p<0.0001, ns = non-significant. To compare antibody binding to HFF cells infected (MOI=3, 96 hpi) with **D,** AD169 or Δ3 and **E,** Merlin or Merlin-Δ4 and **F,** TB40E, followed by the CD16A reporter assay. After supernatant was removed for mIL-2 ELISA, antibody bound to cells was measured by goat-anti human IgG-HRP or goat anti-mouse IgG-HRP ELISA. Data shown are mean ± SD (n=3) from one representative experiment.

Table S1. Pharmacokinetics and thermostabilities of Fc variants

	Fc residue changes	% aggregate after thermal stress	$t_{1/2}$ (days)	Clearance (mL/ day)
**WT**	N/A	2.0 ± 0.8	8.8 ± 0.9	0.30 ± 0.11
**YTE**	M252Y, S254T, T256E	N/A	12.0 ± 2.6 *	0.24 ± 0.09
**IHH**	I253A/H310A/H435A	N/A	0.22 ± 0.01 ****	4.30 ± 0.35 ****
**G5**	S337F, R255Q	ND	5.1 ± 1.4 *	0.43 ± 0.08
**G2**	H268L, E294K, Q311L, K334E, Y407V + R255Q	90.7 ± 0.9***	2.2 ± 0.2 ****	1.26 ± 0.37 ****
**G2E**	H268L, R255Q, Q311L, S337F, and K334E	36.0 ± 2.0***	7.6 ± 0.7	0.32 ± 0.14

Data shown are mean ± SD and compared to WT; statistical significance determined from a one-way ANOVA performed with GraphPad with **p<0.01, ***p<0.001, ****p<0.0001. N/A: not available. For thermal stress, 1 mg/ml protein in PBS was incubated for 20 hrs at 50°C.

Table S2. Oligonucleotides used in Fc yeast display, generation of AD169 mutants, and viral Fc receptor isolation for CHO cell display.

Name	5' to 3' Oligonucleotide	Description
Fc cloning into yeast		
5' hinge CH_2	GGTGGTTCTGCTAGCGACAAAACTCAC	Forward primer for Fc IgG1 cloning into pCTcon2
3' CH_3	ACTGTTGTTATCAGATCTCGAGCTATTACAAGTCCTCTTCAGAAATA AGCTTTTGTTCGGATCCctatttacccggagacagggagaggctct	Reverse primer for Fc IgG1 cloning into pCTcon2
3' CH_2	GGTGTACACCTGTGGTTCTCGGGGCTGCCC	Reverse primer for CH_2 amplification
5' hinge CH_2 long	TACGACGTTCCAGACTACGCTCTGCAGGCTAGTGGTGGTGGTGGTT CTGGTGGTGGTGGTTCTGGTGGTGGTGGTTCTGCTAGCgacaaaactc acacatgcccaccgtgcccagcacct	Forward primer for Fc IgG1 cloning into pCTcon2
3' CH_2 long	ctttgaccaggcaggtcaggctgacctggttcttggtcagctcatcccgggatgggggcagggtgtac acctgtggttctcggggctgccc	Reverse primer for Aga2-CH2
AD169 mutants	-	
KL-DeltaTRL11 Kana1	ACGACGAAGAGGACGAGGACGACAACGTCTGATAAGGAAGGCGAG AACGTGTTTTGCACCCCAGTGAATTCGAGCTCGGTAC	Forward primer deletion of *RL11*
KL-DeltaTRL11-Kana2	TGTATACGCCGTATGCCTGTACGTGAGATGGTGAGGTCTTCGGCAG GCGACACGCATCTTGACCATGATTACGCCAAGCTCC	Reverse primer deletion of *RL11*
KL-DeltaTRL12-Kana1	CGGACGGACCTAGATACGGAACCTTTGTTGTTGACGGTGGACGGG GATTTACAGTAAAAGCCAGTGAATTCGAGCTCGGTAC	Forward primer deletion of *RL12*
KL-DeltaTRL12-Kana1	CCTTACAGAATGTTTTAGTTTATTGTTCAGCTTCATAAGATGTCTGCC CGGAAACGTAGCGACCATGATTACGCCAAGCTCC	Reverse primer deletion of *RL12*
KL-DeltaUL119-Kana1	TTGTTTATTTTGTTGGCAGGTTGGCGGGGGAGGAAAAGGGGTTGAA CAGAAAGGTAGGTGCCAGTGAATTCGAGCTCGGTAC	Forward primer deletion of *UL118-119*
KL-DeltaUL119-Kana2	AGGTGACGCGACCTCCTGCCACATATAGCTCGTCCACACGCCGTCT CGTCACACGGCAACGACCATGATTACGCCAAGCTCC	Reverse primer deletion of *UL118-119*
vFcγR ectodomains	-	
5' AD169 *RL12*	ctgcaaccggtgtacactcgAATAGCACCACAACGA	Forward *RL12*, amplify for display on CHO, c-term flag tag
3' AD169 *RL12*	TCCGCCGCTAGCTGAACCACCTCCCTTATCGTCGTCATCCTTGTAG TCAGATCCACGCGGCAGCTCGAGCCGAGAGCGCTGGCTTGAATG	Reverse *RL12*, amplify for display on CHO, c-term flag tag
5' AD169 *RL11*	caactgcaaccggtgtacactcgAGTTCATCGAACGCCGTCGAA	Forward *RL11*, amplify for display on CHO, c-term flag tag
3' AD169 *RL11*	GATCCGCCGCTAGCTGAACCACCTCCCTTATCGTCGTCATCCTTGT AGTCAGATCCACGCGGCAGCTCGAGTGAGAGGCCGGACCACTGGC GTTTT	Reverse *RL11*, amplify for display on CHO, c-term flag tag
5' AD169 *UL118-119*	actgcaaccggtgtacactcgTCAAGTACAACGAGT	Forward *UL118-119*, amplify for display on CHO, c-term flag tag
3' AD169 *UL118-119*	CGATCCGCCGCTAGCTGAACCACCTCCCTTATCGTCGTCATCCTTG TAGTCAGATCCACGCGGCAGCTCGAGAAGGCGATCCTCGAACAAC GGGT	Reverse *UL118-119*, amplify for display on CHO, c-term flag tag
5' Merlin *RL13*	aactgcaaccggtgtacactcgTATAATCAGACGTGTCC	Forward *RL13*, amplify for display on CHO, c-term flag tag
3' Merlin *RL13*	ATCCGCCGCTAGCTGAACCACCTCCCTTATCGTCGTCATCCTTGTA GTCAGATCCACGCGGCAGCTCGAGGTTCGCTTTTTTAATGGTTT	Reverse *RL13*, amplify for display on CHO, c-term flag tag
